# Beyond CAR-T and oncology: broadening chimeric antigen receptor technologies across cell types and diseases

**DOI:** 10.1093/pcmedi/pbag007

**Published:** 2026-02-23

**Authors:** Xiaohong Liu, Hongye Gao, Jianhua Yu

**Affiliations:** Department of Hematology & Hematopoietic Cell Transplantation, City of Hope National Medical Center, Los Angeles, CA 91010, USA; Department of Hematology & Hematopoietic Cell Transplantation, City of Hope National Medical Center, Los Angeles, CA 91010, USA; Division of Hematology & Oncology, Department of Medicine, School of Medicine, University of California, Irvine, CA 92697, USA; Chao Family Comprehensive Cancer Center, University of California, Irvine, CA 92612, USA

**Keywords:** chimeric antigen receptor, CAR-engineered cells, adoptive cell therapy, immunotherapy, cancer treatment

## Abstract

Chimeric antigen receptor (CAR)-engineered immune cells have revolutionized cancer immunotherapy, expanding from the established success of CAR-T cells to a diverse array of cellular platforms. While seven Food and Drug Administration-approved CAR-T cell products demonstrate unprecedented efficacy in hematologic malignancies, significant limitations persist, including severe inflammatory toxicities, resistance in solid tumors, and manufacturing barriers. These challenges have catalyzed extensive research to extend CAR engineering into alternative effector cell types, such as unconventional T cell subsets, natural killer (NK) cells, macrophages, neutrophils, and dendritic cells, as well as non-immune platforms. Each cell type exhibits distinct antitumor mechanisms, persistence profiles, safety characteristics, and manufacturing requirements, positioning them to address complementary therapeutic needs. This review provides a comprehensive overview of diverse CAR-engineered cellular platforms, encompassing their biological properties, advantages, sourcing strategies, and manufacturing processes, alongside current clinical progress and optimization approaches. Beyond oncology, these platforms have demonstrated significant potential in treating autoimmune diseases, infections, cardiac fibrosis, and senescence-associated disorders. By leveraging distinct immune and non-immune cell types to mediate cytotoxicity or suppress pathogenic cells, CAR technology provides versatile therapeutic avenues across varied disease contexts. Through synthesis of recent advances in CAR platform diversity, this review identifies opportunities for targeted optimization and explores future directions for broadening CAR-based therapeutic applications.

## Introduction

**Figure 1:**
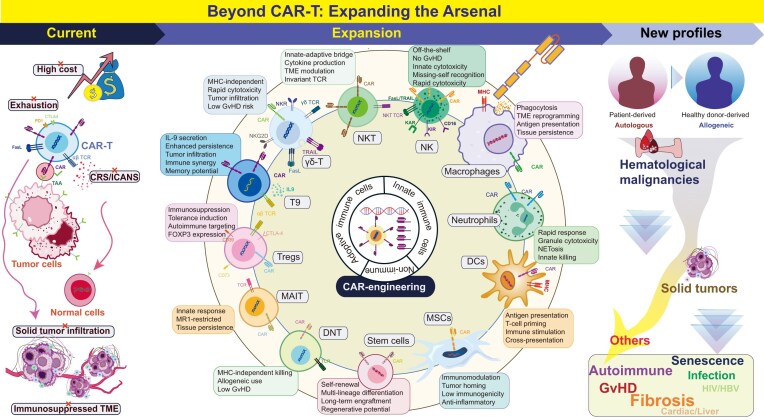
Evolution of CAR technology: from conventional CAR-T cells to diverse cellular platforms and clinical applications. An overview of different CAR cell platforms, outlining their main advantages over conventional CAR-T cells. The flow begins with the major challenges hindering conventional CAR-T cell therapies, such as systemic toxicities, manufacturing hurdles, and resistance in solid tumors. To address these limitations, the field is expanding toward diverse cellular platforms, including various immune effectors and non-immune cells, each offering unique mechanistic advantages. Finally, this diversification facilitates the expansion of therapeutic indications, moving beyond hematologic malignancies to encompass solid tumors, autoimmune diseases, chronic infections, and other diseases.

Chimeric antigen receptor (CAR)-T cell therapy has achieved remarkable clinical success in B-cell and plasma cell hematologic malignancies, resulting in seven products approved by the United States Food and Drug Administration (U.S. FDA), and has emerged as a vital treatment option for hematologic malignancies with expanding indications [1]. Despite impressive outcomes in hematologic malignancies, CAR-T cell therapy faces substantial challenges, particularly in solid tumors. These include limited efficacy stemming from poor trafficking and persistence, antigen heterogeneity, and the immunosuppressive tumor microenvironment (TME). Safety concerns—including cytokine release syndrome (CRS), hematotoxicity, and immune effector cell-associated neurotoxicity syndrome (ICANS) [2]—together with high production costs and requirements for individualized manufacturing, further constrain widespread adoption. In response, researchers are actively refining CAR designs, optimizing manufacturing processes, and extending CAR technology to alternative immune effector cells to overcome these barriers (Fig. 1) [3].

To date, autologous αβ T cells have served as the primary vehicle for CAR engineering [4]. However, to circumvent the inherent constraints of traditional αβ T cell platforms and harness specialized biological functionalities, the research frontier has expanded toward nonconventional T cell subsets and other innate immune cells including γδ T cells [5], regulatory T (Treg) cells [6], IL-9 secreting T (T9) cells [7–9], mucosal-associated invariant T (MAIT) cells, double negative T (DNT) cells, natural killer T (NKT) cells, NK cells, macrophages, neutrophils and dendritic cells [10]. Some studies have even extended CAR technology beyond immune cells to stem cells and mesenchymal stem/stromal cells (MSCs) [11, 12]. Rather than viewing these platforms as interchangeable alternatives, a more sophisticated perspective reveals their distinctive biological attributes, which confer tailored advantages in diverse disease contexts.

Building on these platforms, CAR-engineered cells have also extended their applications beyond cancer immunotherapy [13], demonstrating therapeutic potential in diverse disease contexts, including autoimmune diseases [14], infections [15], cardiac fibrosis [16], graft-versus-host disease (GvHD) [17], and senescence-associated disorders [18, 19]. Leveraging their distinct characteristics, these platforms achieve therapeutic effects through two primary mechanisms: direct elimination or phagocytosing of diseased cells, or suppression of pathogenic cell populations. Specific applications align with these mechanisms. For example, NK cells, which detect infected or senescent cells through a balance of inhibitory and activating receptors [20], exemplify direct elimination and are thus well suited for infectious diseases and age-related conditions. In contrast, CAR-Tregs and CAR-MSCs suppress pathogenic B or effector T cells in autoimmune diseases [21, 22], providing a precise alternatives to conventional systemic immunosuppression. Similarly, CAR-macrophages (CAR-M) remodel the fibrotic microenvironment in cardiac fibrosis through phagocytosis [23]. Collectively, these examples illustrate the versatility of CAR technology beyond oncology, paving the way for broader therapeutic innovations [24].

This comprehensive review synthesizes current knowledge on CAR-engineered cells, emphasizing their properties, advantages, manufacturing processes, and clinical progress. We highlight the challenges these platforms face, along with existing solutions, and discuss comparative considerations across CAR cell platforms in distinct disease contexts, as well as the emerging next-generation CAR therapy ecosystem.

## CAR-αβ T cells: the established gold standard

### Properties and advantages of CAR-αβ T cells

αβ T cells represent the predominant subset of peripheral T lymphocytes, accounting for ∼90%–95% of the total T cell population and are the best-characterized and most extensively studied T cell subset. They express a T cell receptor (TCR) composed of one α chain and one β chain, which specifically recognizes peptide antigens presented by MHC molecules. Based on co-receptor expression, αβ T cells are divided into CD4⁺ helper T cells, which regulate immune responses through cytokine secretion, and CD8⁺ cytotoxic T cells, which directly eliminate infected or malignant cells [25]. Physiologically, αβ T cells are activated through MHC-dependent recognition by antigen-presenting cells such as dendritic cells, which can be recapitulated *ex vivo* to efficiently activate and expand T cells [26].

Conventional CAR-αβ T cells are typically generated as mixtures of CD4⁺ (including Th1 and Th2 subsets) and CD8⁺ T lymphocytes [27]. However, in some studies, these subsets have been evaluated independently, e.g. Boulch et al. and Yang et al. evaluated the functions of CAR-CD4⁺ and CAR-CD8⁺ T cells separately [28, 29]. CD8⁺ T cells exhibit rapid and potent cytotoxicity [30, 31], whereas CD4⁺ T cells contribute to long-term persistence and immunoregulation [32]. Exploiting these complementary features, researchers have optimized CAR-T cell formulations by mixing CD4⁺ and CD8⁺ subsets in defined ratios, enhancing overall therapeutic efficacy [27].

CAR-αβ T cells are engineered to express synthetic receptors combining antibody-derived single-chain variable fragments (scFv) with T-cell receptor signaling machinery. Contemporary second-generation CARs incorporate CD3ζ signaling combined with CD28 or 4–1BB co-stimulation, substantially improving expansion, persistence, and clinical efficacy compared to first-generation designs. Unlike TCR-T cells, CAR-engineered αβ T cells directly recognize cell-surface antigens in an MHC-independent manner; moreover, they exhibit distinct sensitivity and selectivity toward target cells [33, 34]. Upon CAR engagement with a tumor antigen, rapid signaling cascades activate downstream kinases (Lck, ZAP-70), initiating the PLCγ-, PI3K–AKT–mTOR-, and MAPK-dependent signaling pathways. These pathways collectively promote the activation of NFAT, AP-1, and NF-κB, driving robust proliferation and the upregulation of cytotoxic effector molecules (perforin, granzymes) as well as pro-inflammatory cytokines (TNF-α, IFN-γ, IL-2), which together induce target cell death via pathways such as apoptosis and pyroptosis [35–37].

To date, all the U.S. FDA-approved CAR-T cell products are based on αβ T cells. Moreover, analyses of ClinicalTrials.gov data indicate that CAR-T cell therapies account for the vast majority of registered CAR clinical trials [38]. This predominance reflects widespread adoption of αβ T cells as the primary platform for CAR engineering, owing to several key advantages, including (i) well-established biology with decades of immunology knowledge enabling rational CAR design; (ii) robust *ex vivo* expansion (100–1000-fold in 2–3 weeks); (iii) potent cytotoxic capacity through multiple mechanisms (granule exocytosis, death receptor-mediated killing); (iv) demonstrated long-term persistence in responding patients, with detectable CAR-T cells years after infusion [39]; (v) high transduction efficiencies (60%–80% with lentiviral vectors); (vi) established clinical proof-of-concept with seven U.S. FDA-approved products; (vii) amenable to sophisticated CAR designs including bispecific targeting and logic-gated activation; and (viii) extensive clinical experience enabling sophisticated toxicity management and outcome prediction (Fig. 1, Table 1) [40].

**Table 1:** Comparison of different CAR cell platforms.

Category	CAR Platform	Manufacturing and sourcing	Primary biological advantages	Effector mechanisms	Allogeneic potential
Adaptive cells	CAR-αβ T	Complex cycle; autologous-dependent, HSCs, iPSCs	Well-established standard; established memory formation; robust killing	Perforin/granzyme; pro-inflammatory cytokines	Low (requires TCR/HLA editing)
	CAR-T9	Polarization-dependent differentiation	Superior antitumor function, immunomodulation, persistence and exhaustion resistance	IL-9 signal	Low
	CAR-Tregs	Cytokines induction, FOXP3 overexpression, iPSCs	Antigen-specific immune tolerance; non-cytotoxic	Inhibitory cytokines (IL-10, TGF-β); metabolic disruption	Low
	CAR-γδ T	PBMCs, UCB, iPSCs	MHC-independent recognition; natural tumor tropism	Multiple activation; direct lysis; ADCC; antigen presentation	High
	CAR-MAIT	MR1 tetramer-based sorting from PBMCs	MR1-restricted; potential elevated tumor-homing	Produce cytokines; direct cytotoxicity	High
	CAR-DNT	PBMCs	HLA-independent	Direct cytolysis; immunomodulation	High
	CAR-NKT	PBMCs, HSCs, iPSCs	TME infiltration; targets lipid antigens, no GvHD risk	Multiple activation; TAM modulation	High
Innate cells	CAR-NK	PBMCs, iPSCs, UCB, HSCs, cell lines	Low GvHD/CRS/ICANS risk; rapid killing	Multiple activation; degranulation; ADCC; TRAIL-mediated apoptosis,	Excellent
	CAR-M	PBMCs, peritoneal macrophages, UCB, iPSCs, cell lines	Superior solid tumor penetration and modulating capacity	Phagocytosis; TME reprogramming; antigen presentation	High
	CAR-Neutrophils	iPSCs	Superior solid tumor penetration; BBB and BTB penetration	ROS production; NET formation; phagocytosis	High
	CAR-Dendritic cells	PBMCs, iPSCs	Initiation of endogenous immunity	Cross-presentation; priming of naive T cells	Moderate
Non-immune cells	CAR-HSCs/iPSCs	UCB/Yamanaka factors reprogrammed cells	Clinical-grade manufacturing; unlimited source	Multi-lineage differentiation; self-renewal	Excellent
	CAR-MSCs	Isolate from diverse tissues, iPSCs	Paracrine immunomodulation; tissue-repair homing	Secretion of anti-inflammatory factors	Excellent

Collectively, these features establish αβ T cells as the primary and preferred platform for CAR engineering, combining robust biological functionality with well-validated clinical performance.

### Source and manufacturing of CAR-αβ T cells

CAR-αβ T manufacturing typically begins with peripheral blood collection from patients via leukapheresis, followed by T-cell enrichment through positive or negative selection achieving >95% CD3^+^ populations. Although numerous methods are available to activate T cells, anti-CD3/CD28 beads remain the most commonly used approach for GMP-compliant T-cell manufacturing [41]. Following activation, T cells undergo transduction using viral or non-viral methods. All the seven U.S. FDA-approved CAR-T cell therapies utilize either retroviral or lentiviral vectors for CAR delivery [42]. However, viral vector-based approaches carry a potential risk of insertional mutagenesis due to random genomic integration, and cases of malignant transformation have been reported in some CAR-T cell treatments [43–46]. Additionally, viral production is technically demanding and costly [47]. Therefore, researchers are actively exploring alternative strategies such as site-specific integration mediated by gene-editing tools, mRNA-based approaches, lipid nanoparticle (LNP)-mediated *ex vivo* CAR engineering [48, 49], and transposon-based electroporation to achieve faster and safer CAR-T cell manufacturing [50, 51]. Moreover, some studies have suggested that non-viral CAR-T cells may exhibit superior function compared with virally engineered counterparts [52–55].

Upon successful CAR gene delivery, T cells undergo *ex vivo* expansion over 2–4 weeks to generate clinical-scale CAR⁺ T cells, typically achieving 50%–70% CAR expression in the final product. Quality control assessments include confirmation of CAR expression, T cell viability, sterility and endotoxin testing, and potency assays measuring CAR-T cell-mediated tumor cell lysis. Despite robust manufacturing processes, CAR-T cell therapy faces substantial practical limitations. Manufacturing timelines of 2–4 weeks permit disease progression or clinical deterioration in patients awaiting treatment. Moreover, high therapy cost (typically $300 000–$500 000 per patient in the USA) limits accessibility. Autologous manufacturing is inherently variable, as the quality and quantity of collected T cells depend on disease burden, prior chemotherapy, and individual differences in T-cell function. In patients with heavily pretreated disease, such as those who have received prior chemotherapy or radiotherapy, autologous T-cell quality may be compromised, further impacting the feasibility and efficacy of CAR-T cell therapy [56].

To address these limitations, substantial research efforts have focused on developing allogeneic “off-the-shelf” CAR-T cell products. Allogeneic CAR-T cells, derived from healthy donors or stem cell-derived T cells [11], theoretically offer advantages of standardized manufacturing, faster availability, and reduced cost through economies of scale. The primary barriers to generating allogeneic CAR-T cells from healthy donors are the risk of GvHD and host-versus-graft rejection (HvGR) [57]. GvHD arises when infused allogeneic T cells recognize and attack host tissues, whereas HvGR occurs when the host immune system eliminates the infused allogeneic cells. To mitigate these risks, researchers have employed gene-editing approaches, most notably CRISPR/Cas9-mediated knockout of the endogenous TCR (TRAC or TRBC) and the β2-microglobulin genes. TCR-knockout allogeneic CAR-T cells successfully eliminate endogenous TCR alloreactivity while preserving the CAR-mediated antitumor response. Multiple clinical trials have demonstrated that TCR-knockout allogeneic CAR-T cells can be safely administered without causing severe GvHD while maintaining antitumor activity [58–60], highlighting their promise as a broadly applicable platform with potential utility in both oncologic and autoimmune disease settings [14], as well as their capacity to overcome the manufacturing limitations of autologous CAR-T cell therapy, thereby markedly expanding patient access.

An alternative approach to generate allogeneic CAR-T cells involves deriving CAR-T cells from hematopoietic stem cells (HSCs) and induced pluripotent stem cells (iPSCs). iPSC-derived CAR-T cells offer potential advantages of unlimited scalability, standardized manufacturing processes, and potential for cryopreservation and thawing without loss of function. Preclinical studies have demonstrated that iPSC-derived CAR-T cells bearing αβ TCR can achieve robust *in vivo* expansion and antitumor activity comparable to conventional CAR-T cells [61, 62], although clinical evidence remains limited. In parallel, Carrillo et al. developed anti-HIV CAR-T cells from HSCs, which exhibited superior persistence and antiviral effect compared with *ex vivo* transduced CAR-T cells [63]. Collectively, HSCs and iPSCs may represent promising sources for engineered T-cells.

Another feasible source comprises T cells derived from HLA-matched allogeneic hematopoietic cell transplantation (HCT) donors. Geyer et al. reported that CAR-T cells generated from allo-HCT recipients—manufactured from post-transplant recipient leukapheresis products but largely donor-derived due to hematopoietic chimerism—demonstrated *in vivo* expansion and antitumor activity without inducing significant GvHD [64].

Given the challenges associated with ex vivo manufacturing, and in pursuit of truly “off-the-shelf” or universal solutions, many researchers have recently focused on developing *in vivo* CAR-T cell generation strategies [65]. These approaches aim to directly engineer T cells within the patient’s body using viral or non-viral delivery systems [16, 66], thereby eliminating the need for labor-intensive *ex vivo* expansion. Mechanistically, *in vivo* CAR-T cell therapies rely on targeted delivery platforms such as lentiviral vectors, adeno-associated viruses (AAVs), or LNP engineered to selectively transduce circulating or tissue-resident T cells *in vivo*. Advanced designs often incorporate cell type-specific promoters, transient expression systems, or gene-editing components to enhance specificity, minimize off-target effects, and control CAR expression levels [65]. Notably, several preclinical and early clinical studies have already demonstrated the feasibility and therapeutic potential of *in vivo* CAR-T cell generation [65, 67]. For example, Kelonia Therapeutics recently reported early clinical results for KLN-1010, a fully human anti-B-cell maturation antigen (BCMA) *in vivo*–generated CAR-T cell therapy (lentiviral vector-based delivery system targeting CD3), in patients with relapsed or refractory multiple myeloma. KLN-1010 eliminates the need for leukapheresis, individualized *ex vivo* cell manufacturing, and lymphodepleting chemotherapy, thereby potentially broadening access to CAR-T cell therapies. By day 15 post-infusion, CAR-positive cells comprised ∼22%–72% of CD3⁺ lymphocytes in treated patients. Importantly, all three evaluable patients achieved minimal residual disease (MRD) negativity at month 1, highlighting the therapy’s early clinical promise [68].

### Progress of CAR-αβ T cells

CAR technology was first conceptualized at the end of the 1980s by linking the antigen binding portion of an antibody to intracellular signaling domains of the TCR to transfer the specificity of an antibody to T cells [69]. CAR structural design has evolved across multiple generations, from first-generation constructs containing only a CD3ζ signaling domain to second- and third-generation CARs incorporating one or more costimulatory molecules to enhance T-cell expansion, persistence, and antitumor activity [70]. Clinical translation began with early proof-of-concept studies, most notably the 2011 report by June and colleagues in the *New England Journal of Medicine* demonstrating sustained remissions in leukemia [71]. Subsequently, U.S. FDA approval of the first CD19-directed CAR-T cell product in 2017 for B-cell acute lymphoblastic leukemia marked a major regulatory milestone, firmly establishing CAR-T cell therapy as a transformative modality and accelerating its application in the clinic (Table 2).

**Table 2:** Clinical progress of different CAR cell platforms.

Category	CAR platform	Progress (representative clinical examples)	Clinical indications	Safety and limitations	Infiltration	Persistence
Adaptive cells	CAR-αβ T	7 U.S. FDA-approved products	Tumors, autoimmune diseases, infections	High CRS/ICANS risk; exhaustion in solid TME	Low	Moderate
	CAR-T9	Preclinical	Tumors	Limited clinical data; phenotypic stability concerns	Moderate	High
	CAR-Tregs	Clinical stage: NCT04817774, NCT05234190	GvHD, organ transplant, autoimmune diseases	Potential for systemic suppression or plasticity	Moderate	Moderate
	CAR-γδ T	Clinical stage: NCT04735471, NCT06825455	Tumors, autoimmune	Low peripheral frequency; limited persistence	High	Low
	CAR-MAIT	Preclinical stage	Tumors	Low peripheral frequency; nascent clinical evidence	High	Moderate
	CAR-DNT	Clinical stage: NCT06316076,NCT05453669	Tumors, autoimmune	May suppress T cell function	Moderate	Moderate
	CAR-NKT	Clinical stage: NCT03294954,NCT06394622	Tumors	Extremely low frequency in PBMCs	High	Moderate
Innate cells	CAR-NK	Clinical stage: NCT05213195, NCT04324996	Tumors, autoimmune diseases, infections	Short lifespan; cytokine support dependency	Low	Low
	CAR-M	Clinical stage: NCT04660929, NCT05138458	Tumors, autoimmune diseases, fibrosis	Difficult transduction; requires repeated dosing	High	Low
	CAR-Neutrophils	Preclinical stage	Tumors, infections	Very short half-life; requires repeated dosing	High	Very Low
	CAR-Dendritic cells	Clinical stage: NCT05631899, NCT05631886	Tumors	Complex functional maturation	Moderate	Low
Non-immune cells	CAR-HSCs; CAR-iPSCs	Preclinical stage; Clinical stage: NCT03841110, NCT04363346	Tumors, multiple diseases	Potential for insertional mutagenesis; immaturity	Variable	Variable
	CAR-MSCs	Preclinical stage	Autoimmunity	Low immunogenicity; risk of supporting tumor stroma (context-dependent)	High	Moderate

CAR-αβ T cell therapy has achieved unprecedented clinical efficacy in B-cell and plasma cell hematologic malignancies. In B-cell acute lymphoblastic leukemia (B-ALL), CD19 CAR-T cell therapy achieves complete remission (CR) rates of 40%–85% in relapsed/refractory patients [72–74]. In B-cell non-Hodgkin lymphoma (NHL), CR rates of 50%–80% are achieved in relapsed/refractory populations with durable remission in 40%–60% at 2–3 years follow-up [75]. In multiple myeloma, BCMA-directed CAR-T cells achieve response rates >80% with very good partial response or better in 75%–85% of treated patients [76].

Beyond hematologic malignancies, the exploration of CAR-T cell therapy continues in solid tumors and autoimmune diseases. In solid tumors, the overall objective response rate remains modest (typically <30% in most studies) and is lower than that observed in hematologic malignancies [77]. Meanwhile, in autoimmune diseases, early clinical data are highly encouraging: in systemic lupus erythematosus (SLE), autologous BCMA-CD19 compound CAR-T cell therapy has induced symptom- and medication-free remission in some patients, with no severe CAR-T cell-associated toxicities reported in small cohorts (Tables 1 and 2) [78].

### Challenges and optimization of CAR-αβ T cells

#### CRS

The most common serious adverse event occurring in 30%–90% of CAR-T cell-treated patients [79] arises from robust CAR-T cell activation upon tumor encounter, resulting in massive inflammatory cytokine release (e.g. IL-6, TNF-α, IL-2, IFN-γ), with peak serum IL-6 levels exceeding physiologic levels by 100–1000-fold in severe cases [35, 80]. Mild CRS (grade 1–2) manifests as fever and fatigue, typically resolving within 1–3 days [81]. Severe CRS (grade 3–4) presents with high-grade fevers, hypotension requiring vasopressor support, and potential progression to multi-organ failure if untreated [82]. Tocilizumab (anti-IL-6 receptor monoclonal antibody) provides rapid CRS resolution in 80%–90% of cases, enabling CAR-T cells continued expansion and disease control [83]. Corticosteroids represent alternative CRS management for tocilizumab-refractory cases but may reduce CAR-T cell expansion [82]. Given that severe CRS can be life-threatening, some researchers have attempted to reduce its incidence by blocking IL-1 signaling [84], knocking out cytokines such as granulocyte-macrophage colony-stimulating factor (GM-CSF), IL6 and IFN-γ [85–87].

#### Neurotoxicity

ICANS affects 20%–50% of CAR-T cell-treated patients, manifesting as acute encephalopathy with confusion, seizures, and potential cerebral edema [88]. Dexamethasone provides effective first-line ICANS management for mild-to-moderate cases [88]. Management of ICANS remains difficult because of its variable clinical presentation and the requirement for continuous neurological assessment. Mechanistic studies indicate that ICANS is linked to endothelial dysfunction, cytokine-driven blood–brain barrier permeability, and neuroinflammation involving IL-1, GM-CSF, IL-6, and myeloid cell activation within the central nervous system (CNS) [89].

#### Manufacturing limitations

**Figure 2:**
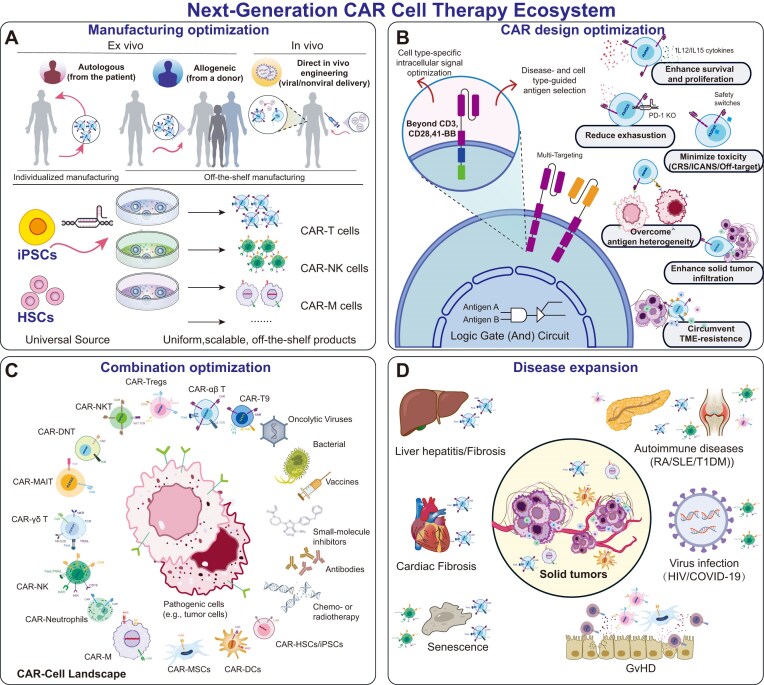
Strategic roadmap for the optimization and functional diversification of CAR-based platforms. Progress in CAR technology is characterized by a multidimensional evolution across manufacturing, molecular design, and clinical application. (**A**) Manufacturing optimization: evolution from individualized autologous workflows to standardized, allogeneic “off-the-shelf” production using iPSCs or HSCs sources, ensuring high scalability and consistency, with emerging in vivo CAR engineering enabling direct modification of endogenous cells in the body. (**B**) CAR design optimization: strategies include rational target antigen selection, implementation of cell type–specific co-stimulation domains, logic-gated circuits, reduction of exhaustion, enhancement of persistence and tumor infiltration, and improvement of target specificity, all aimed at maximizing the efficacy of cell therapies while minimizing off-tumor toxicities. (**C**) Combination optimization: exploiting the complementary biological attributes of diverse treatment methods (e.g. living drugs and non-living drugs) to overcome systemic resistance across various diseases. (**D**) Disease expansion: translation of the CAR paradigm beyond oncology into autoimmune, infectious, and other disease contexts, facilitated by the selection of disease-appropriate cellular chassis.

Autologous manufacturing requires complex GMP facilities, specialized expertise, typically takes 3–4 week timelines, and fails in 5%–10% of cases [56]. Costs exceed $300 000 per patient in many contexts, severely restricting global access [90]. Development of allogeneic CAR-αβ T cells derived from HSCs or iPSCs, *in vivo* CAR-T cell approaches, and other universal CAR-T cells strategies holds considerable promise for significantly reducing manufacturing costs and improving accessibility (Fig. 2A, Table 1) [3, 57].

#### Lymphodepletion

Chemotherapy conditioning enhances CAR-T cell expansion but causes toxicity [91]. Major hematologic toxicities include cytopenias such as neutropenia, leukopenia, anemia, and thrombocytopenia, which can be managed with administration of hematopoietic growth factors [92]. Recent studies indicate that hematologic toxicity is not solely from lymphodepleting chemotherapy but may also result from CAR-T cell infiltration into bone marrow, inducing inflammation and depleting the HSC pool [2]. Therefore, limiting CAR-T cell infiltration into the bone marrow may help reduce this toxicity. Additionally, CD19-targeted CAR-T cells induce profound B cell aplasia necessitating long-term immunoglobulin replacement (monthly intravenous immunoglobulin) and antimicrobial prophylaxis, contributing substantially to infectious complications occurring in 10%–30% of CAR-T-treated patients [93]. *In vivo* CAR-T cell therapy offers the advantage of bypassing lymphodepletion; although challenges remain, it holds considerable potential [65].

#### Efficacy improvement in hematologic and solid tumors

Disease relapse occurs in 20%–60% of initially responding patients, particularly in hematologic malignancies, with antigen-loss escape representing the predominant CAR-T resistance mechanism. Dual-CAR designs simultaneously targeting two antigens may reduce relapse rate [94]. Researchers are developing multi-target CAR-T cell therapies that simultaneously target multiple antigens, combine different CAR-T cell populations, or incorporate logic-gated designs to enhance safety [95]. In solid tumors, major barriers include antigen heterogeneity, poor trafficking to tumor sites, limited persistence, and immune suppression within the TME (Fig. 1). To date, CAR structures have evolved from the first to fifth generation to optimize efficacy, and the optimization strategies focus on multi-antigen targeting or new target developing [96], armored CARs secreting cytokines or checkpoint inhibitors, affinity-tuned and logic-gated CAR designs, and combination regimens with immune checkpoint blockade or cytokine modulation [97]. For example, armored CAR-T cells can be engineered either to secrete cytokines, such as IL-7, IL-21, IL-18, or IL-23, to enhance T-cell expansion, persistence, and effector function [97], or to express tumor microenvironment–modulating factors, including a dominant-negative TGF-β receptor II (TGFBR2-DN) or TNFSF14 (LIGHT), which enhance antitumor efficacy by reshaping the tumor microenvironment [77, 98, 99]. To increase CAR-T cells infiltration into cold tumors, researchers have engineered CAR-T cells to overexpress various chemokines or chemokine receptors such as CCL19, CCL21, CXCR2, or CXCR6 to improve trafficking and extravasation [100–103]. Additionally, targeting the tumor vasculature and stroma, and combining CAR-based therapy with other treatment modalities such as oncolytic virus, may further enhance tumor infiltration [104–107].

#### Exhaustion

CAR-T cell exhaustion arises from persistent antigen stimulation, tonic CAR signaling, immunosuppressive TME, and manufacturing-related factors [108], leading to reduced cytotoxicity and proliferation. Exhaustion is associated with poor clinical responses and is characterized by upregulation of inhibitory receptors (e.g. programmed cell death protein 1 (PD-1), TIM3, CD39) and exhaustion-related transcription factors (e.g. TOX, NR4A) [109]. Recent studies show that T cells in solid tumors can lose function rapidly, as early as 6 h after tumor cells contact [110]. Multiple strategies have been developed to alleviate exhaustion and enhance T-cell persistence. Structural CAR optimization can reduce tonic signaling and delay exhaustion [111]. Additionally, overexpression of beneficial molecules or deletion of inhibitory ones—such as cytokines or transcription factors like c-Jun—can improve CAR-T cell expansion and persistence [112]. Approaches involving T cell stemness regulation, pharmacologic modulation, and metabolic reprogramming have also shown potential to enhance CAR-T cell efficacy [111]. Combined application of these strategies may further improve therapeutic outcomes and expand clinical possibilities for CAR-T cell therapy [113].

#### Safety improvement in hematologic and solid tumors

On-target, off-tumor toxicity represents a major concern in the use of CAR-T cells against malignancies. Although CD19 is an excellent target for hematologic malignancies and certain autoimmune diseases, it is not a true tumor-specific antigen (TSA). CD19 is physiologically expressed on normal B cells and has also been detected in mural cells of the brain [114]. Despite this, CD19-directed therapies exhibit potent antitumor activity with generally manageable on-target, off-tumor toxicity. In contrast, identifying ideal antigens for solid tumors is far more challenging. Very few antigens are exclusively expressed on tumor cells; most are tumor-associated antigens (TAAs) such as CEA, HER2, EGFR, GD2, mesothelin (MSLN), and MUC1 [115]. Although TAAs often exhibit differential expression between malignant and normal tissues, even low-level expression in healthy organs can lead to severe toxicities, exemplified by a fatal case in which a patient with metastatic colorectal cancer died after receiving HER2-CAR-T cells due to low-level HER2 expression on pulmonary epithelium [116]. To enhance CAR-T cell therapy safety, multiple strategies have been developed (Fig. 2B). One approach involves modifying CAR constructs to improve tumor cell recognition while minimizing damage to non-malignant cells. Another strategy is the use of logic-gated CAR-T cells, which employ logical gates such as “OR”, “IF/THEN”, “NOT”, and “AND” to regulate CAR-T cell activation. Additionally, clinically approved drugs can trigger suicide switches, rapidly inducing CAR-T cell self-elimination to control their activity. Acoustic or optogenetic switches, which use ultrasound or light to regulate CAR expression or assembly, provide precise temporal and spatial control. Local CAR-T cell administration is also being explored to overcome challenges associated with systemic delivery [117].

## CAR-γδ T cells

### Properties and advantages of CAR-γδ T cells

Gamma-delta (γδ) T cells, comprising 1%–5% of peripheral lymphocytes, are characterized by expression of TCRs composed of γ and δ chains (in contrast to the conventional αβ TCR expressed by 95%+ of circulating T cells) [118]. γδ T cells occupy an evolutionary position between adaptive and innate immunity, combining elements of TCR-based antigen recognition with innate-like features including NK receptor expression and rapid, non-MHC-restricted cytokine production (Table 1). Human γδ T cells constitute a heterogeneous lymphocyte population that utilizes a diverse repertoire of Vγ (2–5, 8, and 9) and Vδ (1–8) chains to form the heterodimeric γδ TCR [119]. γδ T cells are broadly categorized based on their TCR Vδ-chain usage: Vγ9Vδ2 T cells, which dominate peripheral blood, are activated by phosphoantigens through a butyrophilin-dependent pathway involving BTN3A1 and BTN2A1, and can be selectively expanded *in vitro* and *in vivo* using zoledronate (ZOL), an aminobisphosphonate routinely used in clinical practice [120]. Besides their TCR signals, Vδ2 T cells also express Toll-like receptors (TLRs) and natural killer receptors (NKRs, e.g. NKG2D and DNAM-1). TLRs can respond to pathogen/damage-associated molecular patterns, while NKG2D mediates recognition of transformation-associated stress ligands such as MICA/B and ULBPs [121]. In addition to potent innate cytotoxicity, Vγ9Vδ2 T cells possess antigen-presenting capabilities and can cross-present antigens to conventional αβ T cells [122]. Vδ1 T cells, by contrast, are primarily enriched in epithelial and mucosal tissues and respond to stress-induced or tumor-associated ligands such as MICA/B, ULBPs, and CD1-presented lipids via NKG2D and their TCR [123]. They typically display a more naive-like or stem cell memory-ike phenotype with lower expression of exhaustion markers compared with Vδ2 cells [124]. Additional subsets, including Vδ3 T cells, are found predominantly in the liver and contribute to antiviral immunity and local immune regulation, though they remain less explored for cell-therapy applications [118, 125]. To date, CAR-engineering efforts have focused mainly on the Vδ1 and Vδ2 subsets due to their abundance, accessibility, and favorable functional profiles.

CAR-γδ T cells offer several potential advantages compared to CAR-αβ T cells (Table 1). First, γδ T cells demonstrate an intrinsic ability to infiltrate solid tumors and epithelial tissues, which may enhance access to the TME [126]. Second, γδ T cells display substantially reduced alloreactivity compared with αβ T cells, a property related reduced capacity for MHC-restricted recognition of allogeneic tissues. This reduced alloreactivity suggests potential for allogeneic “off-the-shelf” CAR-γδ T cell products without TCR-knockout necessity. Third, γδ T cells demonstrate innate-like tumor recognition capabilities through expression of NK receptors and ligand recognition patterns, potentially reducing dependency on CAR targeting of single antigens [127].

### Source and manufacturing of CAR-γδ T cells

CAR-γδ T manufacturing involves isolating γδ T cells from peripheral blood via positive selection using γδ TCR-specific antibodies (anti-TCR γδ) or negative selection targeting non-γδ populations, often achieving γδ T enrichment to >80% purity. Following isolation, distinct expansion strategies are applied depending on γδ T cell subset. For Vδ2 γδ T cells, ZOL is the most widely used and robust method. ZOL-induced phosphoantigen accumulation drives potent and selective Vγ9Vδ2 activation, enabling highly efficient and clinically scalable expansion—making Vδ2 cells the most accessible γδ T subset for adoptive cell therapy. For Vδ1 γδ T cells, selective expansion remains more challenging because no cognate Vδ1-specific TCR ligand has been identified. Nonetheless, emerging clinical-grade protocols reported by Almeida and Ferry—using anti-CD3 antibody plus cytokines stimulation, or αβ T cell depletion followed by cytokine-driven expansion—support reliable large-scale Vδ1 manufacturing from peripheral blood and have facilitated the entry of Vδ1-based CAR-γδ T products into early-phase clinical development [128, 129].

More general γδ T cell expansion protocols also exist. Anti–γδ TCR stimulation combined with IL-2 (100–500 U/ml routinely yields 100–2000-fold expansion within 2–3 weeks [130]. Artificial antigen-presenting cell (aAPC) feeder systems can further enhance yield [131]. Recent studies from our group and others have additionally shown that mitogen-based activation (e.g. cncanavalin A) can drive potent Vδ1 T cell proliferation at scale [5, 131, 132].

In addition to peripheral blood, γδ T cells can also be isolated and expanded from umbilical cord blood (UCB) [133]. Although the baseline frequency of γδ T cells in UCB is low, over half of γδ T cells in UCB are of the Vδ1 subset, making UCB a source enriched in less differentiated γδ T cells compared with peripheral blood [134]. With optimized in vitro expansion protocols, even these initially rare UCB γδ T cell populations can be robustly expanded, demonstrating the feasibility of generating clinically relevant numbers of CAR-γδ T cells from cord blood [134, 135].

For genetic engineering, lentiviral vectors remain standard, generating 50%–80% CAR expression efficiency, and the overall manufacturing workflow is highly compatible with established CAR-T cell processes, as γδ T cells tolerate activation, transduction, and expansion conditions similar to those used for αβ T cells. Similarly, analogous to αβ T cell manufacturing protocols, non-viral CAR delivery approaches—such as mRNA electroporation and transposon-based methods—are increasingly explored in CAR-γδ T cell production [124, 136, 137], providing greater manufacturing flexibility and potential reductions in production timelines. Overall, current manufacturing workflows can routinely produce clinically relevant doses of CAR-γδ T cells within 2–3 weeks, supporting their feasibility for translational and therapeutic applications.

### Progress of CAR-γδ T cells

Development of CAR–γδ T cell therapy has progressed significantly across both hematologic malignancies and solid tumors. In B-cell leukemia, preclinical studies using CD19-directed CAR-γδ T cells demonstrated potent *in vitro* and *in vivo* cytotoxicity even in antigen–loss settings: donor-derived γδ T cells engineered with a CD19 CAR showed robust cytokine production (IFN-γ, TNF-α), expansion, and leukemia clearance in xenograft models [138]. Adicet Bio is a leading innovator in CAR-γδ T cell therapy. In their phase I study evaluating ADI-001 for the treatment of B-cell malignancies, the therapy achieved a 78% overall and complete response rate, with no reported cases of GvHD or severe CRS or ICANS [139]. In solid tumors, CAR-γδ T cell engineering has also shown substantial promise. Owing to their intrinsic capacity for tissue infiltration, CAR-γδ T cells are particularly well suited for solid tumor treatment [126]. They can potentially overcome the barriers of tumor evasion through multiple cytotoxic mechanisms. These include CAR and their native TCR signals [129], FasL/TRAIL-mediated apoptosis, and NKG2D-mediated recognition of stress ligands [127, 137]. Furthermore, they can remodel the immunosuppressive TME through the secretion of pro-inflammatory cytokines, and even function as antigen-presenting cells to initiate endogenous antitumor immune responses [132]. For example, in hepatocellular carcinoma, glypican-3 (GPC3) specific CAR Vδ1-T cells co–expressing IL-15 demonstrated enhanced expansion and antitumor efficacy in preclinical models [140]. Moreover, recent translational efforts have proposed dual–target CAR-γδ T cells for neuroblastoma, such as PTK7/GD2 CAR-γδ T cells, aiming to improve tumor-homing and persistence; this strategy is under development for pediatric patients [141]. In addition, numerous studies show that CAR-γδ T cells exhibit strong efficacy and favorable safety profiles across multiple solid tumors, including renal cell carcinoma, prostate cancer, colorectal cancer, lung cancer, oral cancer, and breast cancer [125]. In a phase I clinical trial of CAR-γδ T cells targeting B7H3 for the treatment of glioblastoma, Li et al. demonstrated that among 7 subjects, the objective response rate was 42.9%, with no severe CRS, ICANS, and GvHD observed [142]. Numerous clinical trials investigating CAR-γδ T cell therapy are currently underway, including hematologic malignancies, solid tumors, and autoimmune diseases [143]. Although research on CAR-γδ T cells in autoimmune diseases remains limited, early findings indicate promising “off-the-shelf” therapeutic potential. Notably, a clinical trial is currently evaluating CD19-directed universal CAR-γδ T cells for the SLE treatment (NCT06106893) (Table 2).

### Challenges and optimization of CAR-γδ T cells

One of the major challenges for γδ T cell therapies is the difficulty in obtaining sufficient γδ T cells for therapy. In addition to optimizing expansion protocols [144], using iPSC-derived γδ T cells, as demonstrated by Wallet et al. [145], represents an alternative approach. CAR-γδ T cell persistence remains incompletely characterized across preclinical and clinical trials, and emerging evidence suggests that limited persistence may also represent a potential challenge for this platform [124, 146]. To further enhance the persistence and functionality of γδ T cells, cytokine engineering strategies—such as IL-2 or IL-15 expression—have been employed to improve their long-term antitumor efficacy [140, 147]. In terms of efficacy, in addition to some armored CAR designs [148], current studies have shown that combining CAR-γδ T cells with T-cell engagers (TCEs) or immune checkpoint inhibitors can augment their antitumor activity [149]. Regarding safety, the development of logic-gated CAR-γδ T cells represents a promising approach to modulate their activity through the simultaneous targeting of multiple tumor antigens, thereby reducing off-tumor toxicity and overcoming immune evasion [146]. Building on some overlapping challenges, many of the enhanced engineering strategies originally developed for αβ T cells can likewise be adapted and applied to CAR-γδ T cells (Fig. 2B) [150]. Notably, tumor-infiltrating γδ T cells have been reported to acquire immunosuppressive properties in specific tumor contexts, including suppression of T cell and dendritic cell function via a TLR-controlled signaling pathway, which may limit antitumor efficacy and warrants careful consideration in the design of CAR-γδ T cell therapies [151].

## CAR-Tregs

### Properties and advantages of CAR-Tregs

Tregs, predominantly characterized by CD4^+^CD25^+^FOXP3^+^ phenotype and comprising 5%–10% of peripheral CD4^+^ T cells (primarily αβ T cells), maintain immune tolerance through multiple immunosuppressive mechanisms including immunosuppressive cytokines (particularly IL-10 and TGF-β) and inhibitory molecules expression (e.g. CD39, CD73, cytotoxic T-lymphocyte-associated protein 4 (CTLA-4) and PD-1), and contact-dependent effector T cell suppression (Fig. 1). Tregs employ multiple complementary mechanisms to restrain effector T cell (Teff) activation, proliferation, and cytokine production, thereby maintaining peripheral immune tolerance. First, Tregs secrete suppressive cytokines—including IL-10, TGF-β, and IL-35—that inhibit Teff function and dampen antigen-presenting cells (APCs) activation (Fig. 1, Table 1). Second, they exert contact-dependent suppression through inhibitory receptors such as CTLA-4, LAG-3, TIGIT, and PD-1/Programmed death-ligand 1 (PD-L1), which directly suppress Teff signaling and downregulate costimulatory molecules on APCs. Third, Tregs impose metabolic disruption, most notably by high-affinity IL-2 consumption via CD25, thereby limiting IL-2 availability to Teff. They also generate immunosuppressive adenosine through CD39/CD73 and transfer cAMP to Teff to inhibit NF-κB-dependent activation [152]. In addition, Tregs can directly eliminate Teff or APCs through granzyme- and perforin-mediated cytolysis, and they modulate APC function by inducing tolerogenic programming, including Indoleamine 2,3-dioxygenase (IDO) expression [153, 154].

Engineering Tregs with CAR leverages these intrinsic suppressive pathways while providing antigen-specificity, enabling targeted modulation of pathogenic immune responses. Unlike conventional CAR-T cells designed to eliminate pathogenic cells through potent cytotoxicity, which often results in profound cytopenia and a subsequent reliance on IVIG support to prevent infections [155], CAR-Tregs represent a fundamentally distinct immunotherapeutic platform [21, 156]. Rather than mediating tumor-killing, they exert precise immunosuppression, functioning as a “programmable tolerance inducer” to selectively restrain autoreactive or alloimmune responses while preserving global immune competence [157]. As demonstrated in islet transplantation models, A2-CAR-Tregs induced linked suppression and infectious tolerance, maintaining transplant tolerance even after the CAR-Tregs were no longer detectable [158]. Recently, Moorman et al. developed conventional DC1 (cDC1)-targeted anti-XCR1 CAR-T cells and CAR-Tregs and evaluated their efficacy in the experimental autoimmune encephalomyelitis (EAE) model. Findings indicate that depletion of cDC1 (via CAR-T cells) or immune suppression (via CAR-Tregs) can modestly suppress Th1-driven EAE [159]. These studies reveal a key functional distinction between the two platforms: CAR-T cells are better suited for the efficient elimination of defined pathogenic immune populations, whereas CAR-Tregs appear to be more effective in disease settings where antigen expression is spatially restricted to inflamed tissues, enabling localized immunoregulation and restoration of immune homeostasis.

CAR-Tregs engineering offers theoretical advantages for autoimmune disease therapy: (i) selective targeting of disease-driving cells, enabling localized immunosuppression [160]; (ii) CAR-Tregs preserve their target population, and the antigen exposure could further promote their persistence, expansion, and suppressive activity [161]; (iii) suppression rather than elimination of pathogenic lymphocytes, with the potential to induce long-term tolerance even in the absence of CAR-Treg persistence [158, 162]; and (iv) secretion of predominantly anti-inflammatory, rather than pro-inflammatory cytokines, thereby conferring a favorable safety profile with a reduced risk of CRS [163].

### Source and manufacturing of CAR-Tregs

Tregs are broadly classified into thymus-derived or natural Tregs (tTregs/nTregs) and peripherally induced Tregs (pTregs), reflecting their distinct developmental origins [164]. Notably, Tregs from different sources require tailored expansion strategies. In CAR-Tregs manufacturing, tTregs—which naturally express FOXP3—are most commonly used and are typically isolated from peripheral blood via CD4^+^CD25^+^CD127^low/-^ selection achieving 80%–95% purity [165]. These isolated tTregs are subsequently expanded using anti-CD3/CD28 co-stimulation combined with high-dose IL-2 (500–1000 U/ml) and rapamycin (mTOR inhibitor) supplementation preferentially promoting Treg expansion while suppressing Teff growth, achieving 100–500-fold expansion in 2–5 weeks [166, 167]. CAR expression is generally introduced via lentiviral transduction during or after the activation phase, ensuring stable integration and functional CAR expression in Tregs [168, 169].

By contrast, pTregs—generated from naive CD4⁺ T cells under TGF-β and IL-2 conditions—require additional measures such as retinoic acid or rapamycin to sustain stable suppressive phenotype and prevent reversion to effector phenotypes [170]. Besides naturally occurring Treg subtypes, *in vitro* induced Tregs (iTregs) have also been explored for CAR-Tregs generation. Studies by Fransson et al. and Martin et al. demonstrated that FOXP3 overexpression in conventional CD4⁺ T cells reliably confers a regulatory T cell phenotype, enabling these cells to effectively suppress autoimmune disease progression [171, 172]. These differences between tTregs and pTregs/iTregs hold significant implications for adoptive immunotherapies, as tTregs generally exhibit greater lineage stability and are preferred for clinical-scale expansion [6, 173].

### Progress of CAR-Tregs

CAR-Tregs have been investigated in a wide range of disease contexts, spanning both alloimmune and autoimmune disorders, including GvHD, diabetes, rheumatoid arthritis, multiple sclerosis (MS), inflammatory bowel disease, asthma, vitiligo, hemophilia, and SLE (Table 1) [6, 168]. Preclinical studies have provided strong proof-of-concept for the use of CAR–Tregs in GvHD [174]. Early work by MacDonald et al. demonstrated that aAPC expanded Tregs transduced with a CAR specific for HLA–A2 (A2-CAR) maintain their regulatory phenotype and suppressive function *in vitro*, and, when infused into immunodeficient NOD.Cg-PrkdcscidIl2rgtm1Wjl (NSG) mice together with HLA-A2^+^ peripheral blood mononuclear cells (PBMCs), significantly protect against xenogeneic GvHD compared with control CAR–Tregs [175]. On the clinical side (Table 2), the strong preclinical efficacy has spurred translation: anti–HLA-A2 CAR–Tregs are being tested in clinical trials, including the “STEADFAST” trial (NCT04817774), for kidney transplant recipients and the “LIBERATE” trial (NCT05234190) for liver transplant recipients [176]. CAR-Tregs have also demonstrated considerable therapeutic potential in autoimmune diseases. For example, Doglio et al. demonstrated that FOXP3-overexpressing, CD19-targeted CAR-Tregs effectively reshaped the B cell compartment and reduced inflammation in SLE models, resulting in improved survival and favorable safety [168]. Recently, Sonoma Biotherapeutics reported that their CAR-Tregs product SBT777101 was well tolerated following infusion in rheumatoid arthritis patients, with no cytokine release syndrome, neurotoxicity, or other immune-mediated adverse events observed [177].

### Challenges and optimization of CAR-Tregs

Purification and expansion of Tregs remain significantly challenging due to their very low frequency in peripheral blood. On one hand, autologous CAR-Tregs manufacturing requires isolation of highly purified Tregs using magnetic bead-based selection or flow cytometric sorting. These procedures substantially increase production costs, potentially making the financial burden of CAR-Tregs therapy comparable to that of currently approved autologous CAR-T cell therapies [178]. On the other hand, it is critical during manufacturing to preserve Treg lineage identity and maintain stable FOXP3 expression, as Tregs are prone to losing FOXP3 expression, and acquiring pro-inflammatory effector features under inflammatory or suboptimal culture conditions [179]. Therefore, further optimization of CAR-Tregs production strategies and therapeutic efficacy remains necessary. Developing allogeneic CAR-Tregs or generating Tregs from iPSCs represents a promising alternative. Notably, Yano et al. successfully differentiated iPSCs into CAR-expressing CD4⁺ Treg-like cells that effectively mitigated GvHD in preclinical models [180]. Another key limitation is to select proper targets for CAR-Tregs. If target antigens are also expressed in healthy tissues, CAR-Tregs may become broadly activated, reducing precision and risking nonspecific systemic immunosuppression. To overcome this, several modified CAR-Tregs designs have been proposed to improve specificity and safety [164]. Although CAR–Treg therapy has shown promising suppression of alloimmunity and extended graft survival in various preclinical models, its *in vivo* stability and long-term efficacy remain suboptimal [181]. Recently, Lamarthee et al. conducted a comparative study on the use of CD28 versus 4–1BB signaling domains in CAR-Tregs. They found that 4–1BB tonic signaling limited the *in vivo* expansion and persistence of CAR-Tregs [182], indicating that appropriate co-stimulatory signal choice can enhance Treg suppressive function [173]. Additionally, several potential enhancement strategies could be explored to further boost the suppressive activity of CAR-Tregs, such as cytokines overexpression [156].

## CAR-NKT cells

### Properties and advantages of CAR-NKT cells

NKT cells represent a unique subset of αβ T lymphocytes that co-express NK lineage markers. NKT cells can be subdivided into three major functional subsets, NKT1, NKT2, and NKT17, analogous to Th1/Th2/Th17 polarization observed in conventional T cells [183]. These subsets are defined by differential expression of lineage-defining transcription factors and characteristic cytokine profiles. Human type I NKT cells (also known as invariant NKT, iNKT) characteristically use the Vα24-Jα18 TCR α chain (TRAV10–TRAJ18), paired with a limited TCRβ repertoire (Vβ11 in humans), whereas mice express the analogous Vα14-Jα18 chain (TRAV11–TRAJ18). Despite bearing an invariant αβ TCR, type I NKT cells recognizes self- and microbial-derived glycolipids presented by the monomorphic MHC class I-like molecule CD1d (β2M-associated) [184], which means CD1d gene-directed type I NKT cells have limited toxicity when used as autologous or allogeneic products [185], preserving antitumor activity without promoting GvHD. Type I NKT cells are best characterized and predominantly produce IFN-γ. Type II NKT cells, in contrast, possess diverse TCR repertoires and recognize a broader array of lipid antigens, typically exhibiting IL-4-skewed responses and substantial functional heterogeneity [186]. The NKT17 subset, marked by RORγt expression and robust IL-17A secretion, represents an additional functionally distinct arm of the NKT lineage and contributes to tissue inflammation, TME modulation, and mucosal immunity [187].

NKT cells have characteristics intermediate between NK and T cells [188], thereby integrating functional features of both lineages (Table 1 and 2) [189]. This hybrid phenotype allows CAR-NKT cells to retain the potent cytotoxic and antigen-responsive functions characteristic of CAR-T cells, while also exhibiting “off-the-shelf” applicability and favorable safety profiles typically associated with CAR-NK cells [190]. CAR-NKT cells can destroy target cells via direct and indirect mechanisms; upon TCR stimulation, they rapidly produce IFN-γ, promoting Th1 polarization and activating macrophages and NK cells, while their NK-associated receptors (e.g. NKG2D, DNAM-1, NKp30) enable direct, innate-like tumor killing independent of CAR signaling [189]. The CAR further increases tumor specificity and allows CAR-NKT cells to modulate the immune system by licensing dendritic cells, enhancing conventional T cell priming through CD40L and cytokine crosstalk, and inhibiting immunosuppressive cells, thereby amplifying antitumor immunity [191]. Their CD1d–TCR-dependent and innate-like activation programs provide an additional layer of antitumor activity, enabling them to overcome tumor immune evasion even when CAR signaling alone is insufficient. The comparative study by Rotolo et al. demonstrated that, in the presence of CD1d expression, CAR-NKT cells exhibit significantly stronger antitumor activity than conventional CAR-T cells [192]. CAR-NKT cells show promising efficacy in hematologic malignancies and, due to high infiltration and low anergy in the TME, exhibit particular potential against solid tumors [193]. In a solid tumor study, Zhou et al. reported that CAR-NKT cells displayed superior *in vivo* antitumor activity compared with CAR-T cells, driven by CD1d-mediated remodeling of the TME and enhanced endogenous immune activation in mouse models [194]. Moreover, CD1d-mediated targeting and depletion of CRS-associated macrophages can reduce systemic CRS while preserving strong antitumor activity [189, 195].

### Source and manufacturing of CAR-NKT cells

CAR-NKT cell manufacturing begins with isolation of NKT cells from peripheral blood by apheresis. NKT cells can be isolated by CD1d/α-galactosylceramide (αGalCer) sorting or by anti-iNKT microbeads selection [196, 197]. CD1d-tetramer sorting achieves high purity for invariant NKT cells. Anti-iNKT microbeads selection can achieve higher NKT yield but includes NKT cell subsets with diverse characteristics. Isolated NKT cells are subsequently expanded using anti-CD3/CD28 costimulation with IL-2 supplementation. The manufacturing timeline typically spans 2–3 weeks, yielding expansion kinetics comparable to those of conventional T cells [184]. Alternative approaches utilize CD1d-presenting autologous or engineered cells loaded with αGalCer, providing NKT cell-specific TCR stimulation [197]. Given the extremely low frequency of iNKT cells in human blood (0.001%–1%), CAR-iNKT cells from HSCs were developed. Using a feeder-free differentiation protocol, a 10^6^-fold expansion within 6 weeks was achieved [198]. In addition to anti-CD3/CD28 stimulation and αGalCer-loaded PBMC-based expansion methods, irradiated K562 aAPC systems can effectively support expansion of HSC-derived CAR-iNKT cells [195].

### Progress of CAR-NKT cells

Several CAR-NKT cell products targeting both hematologic malignancies and solid tumors have been developed, with antigen specificities including CD19, BCMA, GD2, and prostate stem cell antigen (PSCA) [199, 200], In hematologic malignancies, CD19-directed CAR-NKT cells represent the most extensively studied constructs [143]. Rotolo et al. demonstrated that CAR19-iNKT cells exhibited higher cytotoxicity than conventional CAR-T cells in preclinical models, and early clinical observations indicated that CAR-NKT treatment did not induce severe adverse events [201]. In solid tumors, GD2-targeted CAR-NKT cells have demonstrated notable antitumor activity, particularly in neuroblastoma. These cells possess intrinsic tumor-homing capacity and have been shown to infiltrate tumor tissues more effectively than conventional CAR-T cells. Moreover, CAR-NKT cells can also target tumor-associated macrophages through CD1d recognition, contributing to broader TME remodeling [184, 199]. Based on these favorable preclinical effects in solid tumors, Heczey et al. advanced anti-GD2 CAR-NKT cells into a phase I clinical trial for neuroblastoma, where the therapy was shown to be safe and capable of inducing objective clinical responses [202]. Compared with conventional CAR-T cells, CAR-NKT cells may exhibit similar or lower toxicity under comparable conditions [202]. In our recent study, we found that PSCA-targeted CAR-NKT cells exhibited antitumor activity against pancreatic cancer. They achieved cytotoxic effects comparable to conventional PSCA CAR-T cells without inducing GvHD [197]. Beyond these established targets, a growing array of tumor-associated antigens—including HER2 [203], MSLN [204], GPC3 [205, 206], CD70 [207], EGFR [208], and B7-H3 [209]—is being evaluated for CAR-NKT redirection, reflecting increasing interest in harnessing NKT cells as a versatile and potentially safer alternative to conventional CAR-T cell therapies (Table 2).

### Challenges and optimization of CAR-NKT cells

CAR-NKT cell manufacturing faces challenges due to NKT cell rarity in peripheral blood, requiring selective enrichment and expansion. Manufacturing consistency may be impacted by variable baseline NKT frequencies in different donors and variable expansion kinetics. Consequently, *in vitro* expansion protocols for NKT cells are still under active investigation in both preclinical and clinical settings. To further enhance the expansion, persistence, and antitumor functionality of CAR-NKT cells, some groups have engineered CAR-NKT cells to overexpress IL-15 [210], which could significantly promote NKT cell persistence and function [211, 212]. To achieve high-yield NKT cells for clinical use, researchers are exploring stem cell-derived sources, including iPSCs and HSCs. Ozaki et al. recently demonstrated that NKT cell-derived iPSCs can be differentiated into NKT cells; however, their system relies on stromal cells [213], and generation of CAR-engineered NKT cells from iPSCs remains poorly studied [203]. As an alternative source, Li et al. generated CAR-NKT cells from HSCs using a feeder-free system, achieving ∼10^6^-fold expansion [195]. Although these HSC-derived CAR-NKT cells broadly resembled PBMC-derived CAR-NKT cells, they exhibited a predominantly CD8⁺ single-positive phenotype with a marked paucity of CD4⁺ single-positive cells. Optimizing culture conditions to preserve a balanced Th1/Th2 functional profile—while avoiding skewing toward either extreme—may further improve the functional quality of CAR-NKT cells function [214]. Another key challenge for allogeneic CAR-NKT cell therapy is that these cells can still be eliminated by host T cells due to mismatched HLA molecules, despite their non-alloreactive nature [199]. However, simply knocking down or knocking out HLA class I would provoke host NK-cell-mediated rejection [215]. To prevent allogeneic cells from being eliminated by NK cells and other immune cells such as macrophages, strategies such as HLA-E or CD47 overexpression have been reported. These approaches can effectively protect the cells and extend their survival *in vivo* [57].

## CAR-NK cells

### Properties and advantages of CAR-NK cells

NK cells represent a distinct population of innate lymphoid cells characterized by the ability to recognize and kill target cells without prior sensitization or TCR engagement. NK cells constitute ∼5%–15% of peripheral blood lymphocytes in healthy individuals. NK cell killing is regulated by a balance between activating signals (through NK cell activating receptors including NKG2D, DNAM-1, and natural cytotoxicity receptors) and inhibitory signals (through killer immunoglobulin-like receptors that recognize MHC class I molecules). This “missing self” recognition pattern—wherein NK cells preferentially kill cells that lack MHC class I expression—provides a mechanistic basis for NK cell recognition of cancer cells, which frequently downregulate MHC to evade CD8^+^ T cell recognition. NK cells can also mediate antibody-dependent cellular cytotoxicity (ADCC) through their Fc receptor (CD16), enabling them to kill antibody-opsonized target cells [216, 217].

CAR-NK cells offer several potential advantages compared to CAR-T cells (Fig. 1, Table 1): First, CAR-NK cells demonstrate reduced alloreactivity, which makes CAR-NK cells particularly attractive for allogeneic “off-the-shelf” manufacturing from healthy donor sources [218–220]. Second, CAR-NK cells demonstrate rapid, potent cytotoxicity against target cells through both CAR-mediated and intrinsic NK mechanisms, potentially offering superior killing kinetics compared to CAR-T cells. Third, CAR-NK cells demonstrate reduced potential for CRS, ICANS, and GvHD compared with CAR-T cells. Fourth, CAR-NK cells demonstrate transient expansion and shorter persistence compared with CAR-T cells [221], which can be viewed as a safety advantage (reduced risk of long-term toxicity and loss of CAR specificity through outgrowth of non-CAR populations) or a disadvantage (potentially requiring repeated dosing to maintain therapeutic effect). Fifth, as already mentioned, CAR-NK cells can be manufactured from multiple sources including peripheral blood, UCB, the NK92 cell line, HSC- and iPSC-derived sources [222], potentially offering greater manufacturing flexibility compared with CAR-T cell approaches. NK cell expansion from peripheral blood can be achieved through stimulation with cytokines (particularly IL-2, IL-15, and IL-21) or co-culture with irradiated feeder cells, and does not require the same level of individual optimization as CAR-T cell manufacturing [58].

### Source and manufacturing of CAR-NK cells

Manufacturing of -NK cells can employ multiple cellular sources, providing flexibility not available with current CAR-T cell approaches [223]. Primary NK cells can be derived from peripheral blood through leukapheresis, selective expansion through cytokine stimulation, or feeder cell co-culture, followed by CAR engineering and further expansion [223]. Alternatively, UCB represents a rich source of NK cells with potentially superior expansion characteristics compared with adult peripheral blood NK cells [224]. iPSC-derived NK cells offer potential advantages of unlimited scalability and standardized manufacturing [225], with preclinical studies demonstrating that iPSC-NK cells can achieve comparable transcriptional signature and antitumor function comparable to primary NK cells [226]. The commercial clonal NK92 cell line has also been used in several clinical trials (Table 1) [227, 228].

The choice of genetic engineering method for CAR-NK cells differs somewhat from standard CAR-T cell approaches. Primary human NK cells are intrinsically difficult to transduce with conventional VSV-G-pseudotyped lentiviral vectors, often yielding only low efficiencies [229], whereas retroviral platforms or alternative pseudotypes such as RD114-TR or baboon envelope consistently achieve markedly higher gene-transfer rates [230, 231]. Although retroviral transduction remains the mainstream method for generating CAR-NK cells, efforts to simplify manufacturing and reduce costs have driven interest in non-viral approaches such as electroporation and LNP delivery [216]. Recently, Andorko et al. reported that *in vivo* generation of CAR-NK cells using targeted virus particles achieved ∼9% CAR-positive NK cells detectable in the spleen [232]. *In vivo* generation of CAR-NK cells provides several key advantages and demonstrates substantial promise for future clinical applications [233].

### Progress of CAR-NK cells

CAR-NK cell development has progressed from preclinical proof-of-concept studies to ongoing clinical trials (Table 2) [234, 235]. Multiple reviews and studies indicate that CAR-NK cells exhibit a lower risk of CRS, neurotoxicity, and GvHD compared with CAR-T cells [236–238]. In a recent comparative study of CAR-NK and CAR-T cells, SLAMF7-directed CAR-NK cells exhibit lower on-target, off-tumor effects on healthy cells compared to CAR-T cells, as their activity could be modulated by inhibitory receptors [239]. In terms of cytotoxicity and persistence, Egli et al. demonstrated that, compared with autologous CAR-T cells, allogeneic CAR-NK cells exhibited lower but quicker killing capacity and reduced persistence both *in vitro* and *in vivo* [221]. Consequently, preclinical studies have focused on engineering CAR-NK cells to enhance their tumor-targeting ability, cytotoxic activity, and *in vivo* persistence, achieving antitumor responses in multiple models including hematologic malignancies and solid tumors [237]. Beyond oncology, CAR-NK cells have also been investigated in autoimmune disease and anti-infectious contexts [240]. In one of our studies, we demonstrated that CAR–NK cells targeting the SARS–CoV–2 spike protein could effectively eliminate infected cells, leading to prolonged survival in murine models [15].

Clinical trials of CAR-NK cells have been initiated in multiple hematologic and solid malignancies [227]. Clinical data suggest that CD19-targeted CAR-NK cell products can be administered safely and exhibit favorable toxicity profiles, with markedly lower rates of severe CRS than CAR-T cell therapy and no reported cases of ICANS or GvHD [241–243]. Prior to this, safety had been first proven in relapsed acute myeloid leukemia patients in the clinic using CAR-NK92 cells [244]. Response rates in early trials appear lower than historical controls of CAR-T cell therapy, though direct comparisons are complicated by differences in patient populations, disease burden, and prior treatment exposure [245].

In solid tumors, multiple clinical trials are currently underway targeting a variety of antigens, including CD70, GPC3, HER2, MSLN, PD-L1, PSMA, ROBO1, and NKG2DL [227]. However, to date, publicly available clinical results remain scarce, as most of these studies are still ongoing [246]. In a recent clinical study, Wang et al. demonstrated that NKG2D-based CAR-NK cells engineered to express membrane-bound IL-15 were safe and well tolerated in patients with colorectal cancer [247]. Among the six treated patients, one achieved stable disease while the remaining five experienced progressive; importantly, no severe CRS or other significant adverse events were observed [247]. Given the encouraging outcomes of CAR-NK cell therapy in hematologic malignancies and its favorable safety profile, researchers are increasingly exploring the potential application of CAR-NK cells for the treatment of autoimmune diseases [240]. Recently, Wang et al. reported a clinical trial using iPSC-derived, CD19/BCMA dual-targeting CAR-NK cells for systemic sclerosis. The treatment achieved robust B-cell depletion, and no CRS, ICANS, or GvHD was observed [248]. Notably, CAR-NK cells may represent a highly promising “off-the-shelf” therapeutic platform with broad applications both in cancer and in non-malignant diseases.

### Challenges and optimizations of CAR-NK cells

Despite the theoretical advantages of CAR-NK cells, several challenges limit their current clinical application [249]. A key mechanistic distinction between CAR-NK cells and CAR-T cells involves their functional persistence and expansion patterns. While CAR-T cells, particularly those expressing memory transcription factors, can persist for years and establish long-lived memory responses in some patients [31, 32], CAR-NK cells demonstrate more transient expansion and persistence, with non-engineered NK cells typically persisting for ∼2 weeks [249], necessitating repeated infusions to maintain therapeutic activity [227]. This transience may reflect the inherent biology of NK cells, which exist as “primary” effector cells without establishing long-lived memory compartments in the way that T cells do. Strategies being investigated to enhance CAR-NK cell persistence include: (i) the generation of cytokine-induced memory-like (CIML) NK cells through preactivation with a combination of IL-12, IL-15 and IL-18. These CAR-engineered CIML NK cells have exhibited longer persistence than conventional CAR-NK cells [250]; (ii) checkpoint inhibitor combinations; (iii) lymphodepletion prior to CAR-NK cell infusion [251]; and (iv engineering of CAR-NK cells to express cytokines such as IL-2, IL-7, or IL-15 [223, 252], as we found CAR-NK cells engineered to express membrane-bound IL-15 demonstrate enhanced persistence and increased antitumor activity *in vivo* [197, 227].

Similar to CAR-T cells, the application of CAR-NK cells in solid tumors faces multiple challenges, including an immunosuppressive TME, limited antigen selection, poor tumor infiltration, and rapid functional impairment [253]. Similar to strategies developed for CAR-T cells (Fig. 2B), safety-enhancing methods such as truncated EGFR and inducible caspase-9 suicide switches have already been evaluated in clinical settings [227, 248], and synNotch-based CAR-NK cell systems have been tested for colorectal cancer [254]. To improve CAR-NK cell infiltration into solid tumors, many of the optimization strategies explored in CAR-T cells are also applicable to NK cells [255], e.g. preclinical studies have demonstrated that forced expression of CCL19, CCR2B, or high-affinity CD16 can enhance NK cell function and accumulation within tumors [256]. NK cells rapidly lose their functional capacity within 24 h after infiltrating the tumor site [257]. Thus, modifying CAR-NK cells to address the immunosuppressive TME presents a promising approach. For example, knockout of TGFBR2 or overexpression of TGFBR2-DN in CAR-NK cells can increase resistance to TGF-β-mediated suppression [258].

As with CAR-T cells, enhancing the efficacy of CAR-NK cells remains a major priority not only in hematologic malignancies and solid tumors but also in autoimmune and other disease settings [248]. Currently, multiple strategies are being explored to improve CAR-NK functionality, including optimization of CAR structure [259]; engineering high-affinity, non-cleavable CD16 [260]; high-throughput CRISPR-based screening to identify novel targets for gene knock-in or knockout [261, 262]; multi-antigens–targeting CAR-NK cells [263]; metabolic reprogramming [264]; combination therapies [251]; and disruption of endogenous checkpoint pathways [265].

Another major challenge for CAR-NK cells is their susceptibility to rapid clearance by host immunity, especially when used in allogeneic settings [266]. Although CAR-NK cells have been shown not to induce GvHD, they can still be recognized and eliminated by the host immune system, leading to allorejection. To address this issue, several effective strategies have already been explored in the development of allogeneic CAR-T cells [57]. Recently, Liu et al. demonstrated that selectively knocking down HLA-ABC using shRNA, together with overexpression of HLA-E and PD-L1, could effectively reduce allorejection [266].

## CAR-M

### Properties and advantages of CAR-M

Macrophages represent a diverse population of myeloid cells with critical roles in tissue homeostasis, inflammation, infection control, and tumor surveillance [267]. Tumor-associated macrophages (TAMs) exist in diverse functional states, with the classical pro-inflammatory (activation) phenotype contrasting with the anti-inflammatory (immunosuppressive) phenotype [268]. In contrast to T cells and NK cells, which infiltrate solid tumors inefficiently and rapidly undergo exhaustion or dysfunction within the hostile TME, solid tumors naturally harbor abundant macrophages, which can constitute up to 50% of the immune cell population [269]. These cells are continuously recruited by tumor- and stroma-derived chemokines, yet most become skewed toward anti-inflammatory or immunosuppressive phenotypes that facilitate immune evasion, tumor progression, and metastatic dissemination [268, 270]. Despite this predominant anti-inflammatory polarization, TAMs retain substantial phenotypic plasticity and can be reprogrammed toward pro-inflammatory, tumoricidal states when exposed to appropriate cues, including pattern-recognition receptor signaling, cytokine stimulation, or therapeutic genetic re-engineering [267, 271]. Notably, TAMs are relatively long-lived compared with infiltrating lymphocytes; they can persist for several weeks within tumors and may include self-renewing tissue-resident macrophage lineages [272, 273]. This inherent high penetration and long-term intratumor persistence highlight their unique potential as a vehicle for next-generation immunotherapies in solid tumors.

Engineering macrophages with CARs offers a mechanistically distinct approach to cancer immunotherapy compared to CAR-T or CAR-NK cells [274]. Rather than acting primarily through direct cytolysis, CAR-M mediate antitumor effects through phagocytosis, ADCC, and trogocytosis, and production of pro-inflammatory cytokines, reactive oxygen species (ROS), and nitric oxide [275]. These distinct mechanisms confer additional advantages (Fig. 1, Table 1). On one hand, phagocytosis allows CAR-M to eliminate tumor cells through pathways different from direct cytolysis, potentially overcoming certain resistance mechanisms. Moreover, by presenting phagocytosed tumor antigens to T cells (antigen cross-presentation), they can facilitate the generation of adaptive immune responses [276]. On the other hand, CAR-M can remodel the immunosuppressive TME through the secretion of pro-inflammatory cytokines (IL-12, TNF-α, IL-6) and the expression of costimulatory molecules, potentially shifting the immune balance toward a pro-inflammatory state [277]. Together, these features make CAR-M particularly promising for solid tumors, where their combination of superior infiltration and TME-modulating capacity provides advantages not as readily achieved with CAR-T or CAR-NK cells [278].

### Source and manufacturing of CAR-M

CAR-M generation requires initial procurement of macrophage sources followed by CAR transduction. Viable macrophage sources include: peripheral blood-derived monocytes, peritoneal macrophages [279], cord blood-derived monocytes, commercial macrophage cell lines, and increasingly, iPSCs-derived macrophages (Table 1) [275]. Peripheral blood monocyte-derived macrophages represent the most accessible source currently employed in early CAR-M development [280]. Monocytes isolated via apheresis undergo differentiation into macrophages in culture over 5–7 days with macrophage colony-stimulating factor (M-CSF) or GM-CSF supplementation, generating macrophages with varying pro-inflammatory and anti-inflammatory phenotypes depending on differentiation cytokine selection and culture conditions [280, 281]. Current manufacturing protocols preferentially employ M-CSF differentiation to promote pro-inflammatory macrophage generation [280, 282], followed by engineering of the cells to express the CAR construct. However, primary macrophages possess intrinsic resistance mechanisms to lentiviral transduction [280], resulting in generally low transduction efficiencies (Table 2) [283]. Alternatively, adenoviral-based vectors such as Ad5f35 have been reported to achieve ∼80% positivity. Furthermore, non-viral approaches such as LNP delivery can also be used to engineer macrophages [284]. Notably, Wang et al. successfully generated CAR-M directly *in vivo* using LNP [285]. This strategy offers a safer and more cost-effective alternative, with the potential to enable truly “off-the-shelf” CAR-M products. Recently, Zhou et al. employed enucleated mesenchymal stem cells to deliver CAR-expressing plasmids specifically to macrophages within gliomas. Compared with LNP-based approaches, this strategy achieved higher CAR positivity and enhanced antitumor activity [286]. Compared with primary monocytes, macrophage cell lines and iPSCs are much easier to transduce and genetically modify. We and others have successfully generated CAR-M cells from engineered iPSCs [287–290], and we found that iPSC-derived CAR-M cells phenotypically closely resemble their counterparts from PBMC-derived macrophages. iPSC-derived macrophages represent a potentially transformative manufacturing approach, enabling standardized, “off-the-shelf” production from banked iPSCs with standardized differentiation (2–4 week iPSCs to macrophages differentiation) and minimal batch-to-batch variability [289]. However, iPSCs manufacturing may face regulatory hurdles requiring further validation demonstrating functional equivalence to monocyte-derived macrophages.

### Progress of CAR-M

To enable CAR-M-mediated antigen-specific phagocytosis, Morrissey et al. first developed murine anti-CD19 CAR-M incorporating a panel of distinct cytoplasmic signaling domains, and identified Megf10, FcRγ, and CD3ζ as intracellular modules capable of effectively triggering CAR-dependent phagocytosis for tumor cells [282]. This pioneering study established the foundation of macrophage-based CAR cell therapy. Building on this work, Klichinsky et al. validated the feasibility of CAR-M in both the human THP-1 macrophage cell line and primary macrophages, demonstrating robust antitumor activity in solid tumors. Human HER2-targeted CAR-M were shown to efficiently infiltrate tumor tissue, present antigens, and remodel the TME [280]. More recently, they further demonstrated that CAR-M cells can potently activate endogenous T cells within solid tumors, and that combining CAR-M therapy with PD-1 blockade significantly augments antitumor efficacy [291].

To further enhance CAR-M polarization toward a pro-inflammatory phenotype and improve cytotoxic function, Lei et al. engineered CAR-M incorporating the intracellular TIR domain of TLR4, resulting in markedly enhanced antitumor activity [290]. In parallel, Shen et al. systematically optimized CAR architectures for iPSCs-derived CAR-M and similarly demonstrated their strong therapeutic potential [289]. In our own work, we engineered iPSCs to express a PSCA-targeting CAR together with truncated EGFR (tEGFR) and membrane-bound IL-15, and subsequently differentiated them into CAR-M cells [287]. The inclusion of the tEGFR safety switch enables rapid abrogation of effector function, thereby improving the controllability and overall safety profile of the product [218]. Importantly, neither our studies nor those of others have reported CRS or other severe toxicities in preclinical mouse models [287, 290]. Collectively, these studies establish CAR-M as a highly promising modality for the treatment of both solid tumors and hematologic malignancies [292].

Encouraged by these preclinical data, CAR-M therapies are entering early clinical testing (Table 2) [275, 292]. In the first-in-human phase I trial of CAR-M (Carisma Therapeutics, CT-0508, NCT04660929), CAR-M were shown to infiltrate the TME, remodel the TME, and enhance adaptive immune responses. Among the 14 treated patients, 3 achieved stable disease as a best overall response. Importantly, no dose-limiting toxicities and no severe CRS or ICANS were observed, thereby supporting the acceptable safety profile and emerging therapeutic potential of CAR-M therapy [293]. Beyond the CT-0508 first-in-human study, several additional clinical programs are actively evaluating CAR-M and related myeloid-engineered cell therapies to further establish their safety and efficacy. These include CT-0525 (NCT06254807), an autologous CAR-monocyte product currently in phase I testing; MT-101 (NCT05138458), an mRNA-engineered CAR-myeloid therapy under phase I/II evaluation in CD5⁺ T cell lymphoma; and MCY-M11 (NCT03608618), an earlier phase I study exploring mesothelin-targeted CAR-myeloid cells. Collectively, these ongoing clinical trials highlight the growing momentum of CAR-M-based immunotherapies and continue to generate important evidence supporting their clinical safety and therapeutic potential [292].

Beyond cancer treatment, CAR-M therapies have also shown considerable potential in cardiovascular diseases [294], autoimmune disorders [295], inflammatory diseases [296], liver fibrosis [297], and even anti-aging applications [298, 299]. Recently, Zhou et al. reported that CAR-M can modulate intervertebral disc homeostasis. By using a microneedle array to deliver CAR-M into the deep layers of intervertebral discs, they were able to eliminate apoptotic nucleus pulposus cells, ameliorate the inflammatory microenvironment, and promote disc repair [300]. These findings remain at the preclinical stage, and further investigation is required to translate them into clinical applications [301].

### Challenges and of CAR-M

A critical challenge in CAR-M development involves maintaining a pro-inflammatory macrophage throughout manufacturing and *in vivo* persistence [302], with potential for phenotypic skewing toward anti-inflammatory phenotypes under certain conditions. TME factors (e.g. IL-10, TGF-β) may promote this functional reprogramming *in vivo*, thereby attenuating CAR-M antitumor activity. Optimization strategies include manufacturing conditions maintaining pro-inflammatory phenotype [280, 303], CAR designs promoting sustained pro-inflammatory signaling [290], and co-infusion with cytokines that support inflammatory effector functions (such as IFN-γ) [289].

A key issue limiting the clinical translation of CAR-M therapy is their limited *in vivo* persistence, with early clinical data suggesting CAR-M-mediated antitumor effect was transient and patients progressed within months [293]. Optimal persistence characteristics are required for sustained antitumor function, particularly in solid tumor contexts requiring long-term disease control. Mechanistic studies of factors influencing CAR-M persistence, optimization of CAR designs, and repeated CAR-M administration to enhance persistence represent important future directions [293, 304]. However, unlike lymphocytes, macrophages exhibit minimal proliferative capacity *in vivo* or *ex vivo* [293, 305], making large-scale expansion difficult and necessitating the administration of high cell doses to achieve therapeutic efficacy. In this context, iPSC-derived CAR-M offer substantial advantages by enabling scalable, standardized manufacturing and the generation of sufficient cell numbers. Nevertheless, iPSC-based products carry an inherent tumorigenicity risk [306], which can be mitigated by incorporating safety switches—such as tEGFR or inducible caspase-9—to enable selective elimination of the infused cells and further enhance clinical safety [287, 307]. In addition, recent studies have reported a TME responsive CAR-M platform in which CAR expression is driven by the arginase 1 promoter that targets CD47, providing an alternative and feasible strategy to further improve the safety profile by restricting CAR activity to the TME [308].

Similar to the challenges encountered with CAR-T cell therapy, CAR-M also faces obstacles such as suboptimal tumor infiltration, an immunosuppressive TME, and antigen heterogeneity (Fig. 1 and Fig. 2B). To enhance CAR-M antitumor efficacy, several combination strategies have been explored. For example, co-administration of CAR-M with CD47-blocking antibodies or PD-1 checkpoint inhibitors has been shown to significantly augment antitumor activity [291, 309]. Moreover, combination therapy with CAR-T cells has been reported to produce synergistic antitumor effects (Fig. 2C). Notably, this synergy appears to be bidirectional: CAR-M can enhance CAR-T cell activation, while cytokines produced by activated CAR-T cells promote CAR-M polarization toward a pro-inflammatory phenotype, thereby further strengthening antitumor responses [310]. In addition, CRISPR/Cas9-based functional screening has also been leveraged to optimize CAR-M performance. Using this approach, Wang et al. identified aconitate decarboxylase 1 (ACOD1) as a key target whose modulation markedly enhanced the antitumor activity of CAR-M cells [311]. In parallel, a variety of potential enhancement strategies are currently under active investigation and development to further improve the safety, efficacy, and durability of CAR-M-based therapies [312].

## Other CAR cells: CAR-MAIT cells, CAR-double negative T cells, CAR-T9 cells, CAR-neutrophils, CAR-dendritic cells, CAR-stem cells, and CAR-MSCs

### CAR-MAIT cells

MAIT cells, comprising 1%–10% of peripheral blood lymphocytes (up to 30%–50% in mucosal tissues like the lungs and gut), express a semi-invariant TCR (Vα7.2-Jα33 in humans) recognizing vitamin B metabolites presented by MHC-related protein 1 (MR1) [313]. Similar to NK and NKT cells, MAIT cells can be activated through both TCR-dependent and cytokine-mediated, TCR-independent mechanisms, highlighting their therapeutic potential in both malignant and non-malignant diseases (Table 1) [313]. MAIT cells can be isolated using Vα7.2-based magnetic selection or MR1 tetramer-based sorting, followed by *ex vivo* expansion with cytokine supplementation and the MR1 ligand 5-(2-oxopropylideneamino)-6-D-ribitylaminouracil (5-OP-RU) [313, 314]. Expanded MAIT cells can then be genetically modified using lentiviral vectors in a manner analogous to conventional CAR-T cell engineering. Preclinical studies have demonstrated that CAR-MAIT cells exhibit potent antitumor activity, mediate MHC-independent killing, and secrete lower levels of cytotoxic factors compared with conventional T cells, supporting their potential as a safe and allogeneic platform for CAR-based immunotherapy [314]. Compared with conventional CAR-T cells, MAIT cells uniquely express high levels of chemokine receptors, endowing them with enhanced tissue-homing and infiltration capacities and rendering them particularly attractive for solid tumor immunotherapy [315]. Despite these advantages, CAR-MAIT therapies have not yet advanced to clinical evaluation (Table 2), and challenges related to their limited peripheral abundance and functional optimization remain to be addressed [316].

### CAR-DNT cells

DNT (CD3^+^CD4^−^CD8^−^) cells, comprising 2%–8% of peripheral blood, exhibit rapid activation, intrinsic NK-like killing, and reduced GvHD potential [317]. DNT cells express a diverse TCR repertoire (comprising both αβ and γδ TCRs, with γδ TCR-expressing cells representing the predominant population) [318]. Notably, DNT cells can be efficiently expanded *ex vivo* using standard T cell expansion protocols. Importantly, adoptive transfer of DNT cells neither induces GvHD nor mediates cytotoxicity against normal allogeneic PBMCs or hematopoietic stem cells in murine models [319], highlighting their favorable safety profile. Owing to these properties, DNT cells represent an attractive platform for the development of “off-the-shelf” cellular immunotherapies. Nevertheless, a potential concern remains regarding their lineage stability [320]. As DNT cells have been reported to regain CD8 expression *in vivo* in murine models [320], such phenotypic conversion could theoretically trigger GvHD. Further investigation is required to clarify their clinical relevance and ensure safety. Preclinical studies have demonstrated robust antitumor activity of DNT cells across multiple hematologic malignancy models, including acute myeloid leukemia [319], B-cell malignancies [318, 321, 322], and T-cell malignancies [323]. Clinically, a first-in-human phase I study evaluating CD19-CAR-DNT therapy (RJMty19) reported encouraging safety and preliminary efficacy. In the high-dose cohort, patients in the dose level 4 group (*n* = 3) achieved an overall response rate of 100%, with a complete remission rate of 33%. Importantly, no ≥grade 3 CRS or ICANS were observed, and neither dose-limiting toxicities nor GvHD were reported [324]. Despite these encouraging results in hematologic malignancies, the therapeutic potential of DNT cells in solid tumors and other disease settings remains largely unexplored (Table 2). Notably, a clinical case report has suggested that DNT cells may interfere with the therapeutic efficacy of CAR-T cells [325], and DNT cells have also been reported to suppress T cell function [322, 326]. While such immunomodulatory properties may be advantageous in reducing host-mediated rejection of DNT cells, their broader impact on different disease and combination therapies warrants further investigation [322].

### CAR-T9 cells

Accumulating evidence suggests that IL-9 and IL-9-producing cells exert various roles in antitumor immunity [327]. IL-9 can be produced by various T cell subsets, including T helper 2 (Th2), Th17, Vδ2 T cells, NKT cells, Tregs, CD4^+^ Th9, and IL-9 secreting cytotoxic CD8^+^ T (Tc9) cells [327, 328]. Among these, substantial evidence indicates that Th9 and Tc9 cells possess potent immunoregulatory and antitumor activities [8, 329]. IL-9 secreting T cells (T9) could be differentiated from naive T cells under IL-4 and TGF-β/IL-1β stimulation [330], with transcription factors including PU.1 and IRF4, among others, contributing to the regulation of IL-9 production [331]. It has been reported that TCR- or CAR-engineered Th9 cells exhibit superior antitumor efficacy in murine tumor models compared with Th1, type-I cytotoxic T cell (Tc1)/cytotoxic T lymphocyte (CTL), or Th17 cells [8]. Similarly, Tc9 cells demonstrate reduced exhaustion and enhanced effector function relative to Tc1 cells [332]. Consistent with these findings, recent studies have shown that IL-9 signaling possesses strong pro-inflammatory activity, and that enforced expression of the IL-9 receptor in CAR-T cells markedly improves their expansion, persistence, and tumor infiltration [333, 334]. In addition, CAR-T cells polarized under Th9-culture conditions display increased memory potential and reduced exhaustion [7]. In our recent work, we further identified that ablation of the mRNA reader YTHDF2 promotes Th9 differentiation. Moreover, YTHDF2 depletion in CAR-Th9 cells enhances immune activation, limits terminal differentiation, and augments antitumor efficacy [9]. Although CAR-T9 cell therapies have not yet been evaluated in clinical settings, accumulating evidence indicates that T9 cells possess several advantageous properties, including enhanced persistence, immunomodulatory capacity, improved infiltration into solid tumors, and increased resistance to exhaustion (Tables 1 and 2). Collectively, these features support T9 cells as a promising cellular platform for next-generation cancer immunotherapy [327].

### CAR-neutrophils

Neutrophils, the most abundant circulating leukocytes, represent a potentially valuable population for cell-based therapies [335]. They exhibit tumor-homing properties reminiscent of macrophages and are capable of traversing physiological barriers such as the blood–brain barriers and blood–testis barriers (BBB and BTB), as well as infiltrating hypoxic regions of tumors, making them particularly suitable for drug delivery [336]. Within the tumor microenvironment, infiltrating neutrophils can polarize into distinct functional phenotypes: N1 neutrophils, characterized by high expression of pro-inflammatory cytokines and ligands (e.g. TNF-α, CD86, FAS), exhibit antitumor activity through mechanisms including phagocytosis, production of ROS, release of granule contents such as proteases and antimicrobial peptides, and formation of neutrophil extracellular traps (NETs). In contrast, N2 neutrophils express immunosuppressive molecules such as arginase and PD-L1, thereby inhibiting T cell function (Table 2) [335, 337]. Building on this understanding, Chang et al. explored the development of CAR-modified neutrophils [338]. A key challenge in neutrophil engineering has been their short lifespan (typically 6–8 h in circulation) and the difficulty of sustaining *ex vivo* culture and genetic modification [339, 340]. To overcome these limitations, they utilized iPSC-derived neutrophils and demonstrated that CAR-neutrophils could specifically target solid tumor cells via multiple effector mechanisms, including phagocytosis, ROS production, and NET formation [338, 341, 342]. In addition, the cytotoxic activity of CAR-neutrophils may be less pronounced than that of CAR-T cells, which is at least partly attributable to the inherently short lifespan of neutrophils. Current studies indicate that even with repeated infusions, CAR-neutrophils have not consistently achieved complete tumor eradication in murine models (Table 2) [338]. Moreover, their tumor-infiltrating capacity, *in vivo* persistence, and overall safety profile remain to be fully characterized. Notably, CAR-neutrophils could produce more IL-6 after tumor stimulation, raising concerns regarding a potential risk of CRS [338]. In parallel, neutrophil-mediated effector functions may introduce additional risks of toxicity, underscoring the need for further investigation [343].

### CAR-dendritic cells

Dendritic cells (DCs), professional antigen-presenting cells comprising 0.1%–1% of peripheral blood, efficiently capture and present tumor antigens via both MHC class I and II pathways, secrete immunomodulatory cytokines, express high levels of costimulatory molecules, and bridge innate–adaptive immunity. DCs comprise developmentally distinct populations encompassing monocyte-derived DCs (moDCs), conventional DCs (cDCs), and plasmacytoid DCs (pDCs). cDCs can be further resolved into cDC1 and cDC2 subsets. Among these, cDC1 exhibit superior cross-presentation and cross-priming capabilities and are increasingly implicated in orchestrating antitumor immunity by priming both CD8⁺ cytotoxic T cells and CD4⁺ helper T cells [344, 345], whereas cDC2 primarily present antigens to CD4⁺ T cells and preferentially promote Th2 immune responses [346]. Therefore, harnessing DCs to activate the immune system represents an effective immunotherapeutic strategy. DC vaccines generated by loading defined TAAs or tumor lysates have been shown to efficiently induce antigen-specific immune responses *in vivo* and mediate antitumor activity [347]. In addition, various vaccines encoding TAAs or peptides, including DC vaccines, have been shown to further potentiate CAR-T cell activation and enhance antitumor efficacy [348]. Several studies have demonstrated that CAR-T cells can be effectively activated and expanded upon stimulation with tumor related antigen-pulsed or antigen-loaded DCs [349, 350], highlighting the strong synergistic potential of DC vaccines in combination with CAR-T cell therapy (Fig. 2C). Vaccine-boosted CAR-T cell therapy promoted the activation of DCs and endogenous immune responses, enabling the elimination of heterogeneous tumor cell populations and enhancing immune infiltration within solid tumors [350–352]. Clinically, a nanoparticulate RNA vaccine (CARVac) administered in combination with CLDN6-targeted CAR-T cells has shown encouraging therapeutic activity in patients with solid tumors [353]. Similarly, in hematologic malignancies, this vaccination strategy has been reported to enhance the persistence and antitumor efficacy of CD19-directed CAR-T cells [354]. Building on these advances, engineering DCs with a CAR represents a promising strategy to enable DCs to directly capture tumor antigens while simultaneously processing and presenting these antigens to T cells. In this way, CAR-engineered DCs may combine direct tumor recognition with the coordinated activation of adaptive T cell responses. Recently, Duan et al. demonstrated that CAR and TNF-α engineered DCs can induce the death of cancer cells in the presence of an inhibitor of apoptosis protein (IAP) antagonist [355]. In parallel, the development of an extracellular vesicle-internalizing receptor has been shown to enhance DC uptake and presentation of tumor antigens [356, 357]; however, these strategies do not incorporate intracellular signaling domains required for active DC activation. More recently, Mohammadzadeh et al. have systematically screened CAR signaling modules capable of activating DCs and identified a synthetic immunoreceptor (iCAR) composed of a CD40 activation domain combined with an FcRγ immunoreceptor tyrosine-based activation motif (ITAM), which effectively promotes tumor antigen recognition and initiates robust T cell immune responses [358]. In the context of solid tumors, DC engineering therefore represents a potentially powerful approach to reshape and reprogram antitumor immunity [359]. At present, a representative EphA2-targeting CAR-DC vaccine loaded with a KRAS mutant peptide (KRAS-EphA2-CAR-DCs) is undergoing clinical evaluation in both solid and hematologic malignancies (NCT05631899 and NCT05631886) [360]. Nevertheless, this approach continues to face practical challenges, particularly with respect to scalable manufacturing, and further investigation is therefore warranted (Table 2) [361]. In this regard, iPSC-derived DCs may represent a promising future direction to enable standardized and scalable DC-based immunotherapies [362].

### CAR-stem cells

Stem cells possess several advantageous properties, including self-renewal capacity, multilineage differentiation potential, and amenability to genetic manipulation, which together enable them to overcome some key limitations associated with primary immune cells [363]. Importantly, stem cell-based platforms allow for clinical-grade and standardized manufacturing, with the potential to substantially reduce the cost of current cell therapies [364]. Leveraging these advantages (Table 1), CAR-engineered immune cells derived from HSCs, including CAR-T [63], CAR-NK [365], and CAR-NKT cells [195], are actively being explored and evaluated preclinically; however, to date, these approaches have not yet advanced into clinical testing [366].

Another major stem cell source is iPSCs, which provide an essentially unlimited reservoir for the generation of allogeneic, “off-the-shelf” CAR-engineered cell products [306]. Although differentiation strategies vary considerably among immune lineages and the developmental programs for certain cell types remain incompletely defined [306], iPSCs are capable of generating a broad spectrum of immune as well as non-immune cell types [367]. Beyond approaches that focus on differentiating CAR-HSCs into a single immune effector population, recent studies have also explored the direct therapeutic use of CAR-HSCs [368–370]. In these settings, infused CAR-HSCs were shown to give rise *in vivo* to multiple CAR-expressing immune cell lineages that significantly elicited antitumor activity [370]. Beyond cancer, researchers have demonstrated that CAR-modified hematopoietic stem/progenitor cells (HSPCs) can differentiate into multilineage CAR-expressing immune cells capable of conferring protection against HIV infection and suppressing viral replication *in vivo* [371, 372]. Nevertheless, this strategy may be associated with potential risks. For example, the generation of CAR-expressing B cells could lead to antigen masking or resistance to immune-mediated killing, thereby compromising therapeutic efficacy [373]. In addition, the differentiation of CAR-Tregs or anti-inflammatory CAR-M populations may suppress antitumor immune responses. Furthermore, the use of stem cells raises concerns regarding tumorigenicity, necessitating careful genomic assessment, such as next-generation sequencing, to exclude oncogenic alterations (Table 2). Accordingly, the incorporation of safety switches remains an essential component to enable timely control or elimination of engineered cells when required [374].

### CAR-MSCs

MSCs are emerging as a unique platform that bridges regenerative medicine and cancer immunotherapy. MSCs represent a heterogeneous population of multipotent stromal cells that constitute ∼0.001%–0.01% of bone marrow mononuclear cells and can be isolated from diverse tissues, including adipose tissue and umbilical cord [375, 376]. Beyond their multipotent differentiation and self-renewal capacities, MSCs exhibit low immunogenicity and intrinsic immunomodulatory properties, rendering them particularly suitable for allogeneic “off-the-shelf” cell therapy applications and drug delivery platforms (Table 1). Owing to their ability to suppress both innate and adaptive immune responses, the clinical indications of MSCs have been expanded to GvHD, autoimmune disorders, and other immune-mediated conditions, with several MSC-based products already approved for clinical use in certain countries [377].

Another key function of MSCs is their capacity to promote hematopoietic reconstitution. Recent studies have shown that MSC administration can facilitate HSC recovery and alleviate CAR-T-associated cytopenias without compromising CAR-T cell activity or antitumor efficacy [378]. In addition, MSCs possess intrinsic tumor-homing properties; within solid tumor settings, they can be actively recruited by chemokines secreted by the TME. Leveraging this feature, MSCs have been explored as targeted delivery vehicles for therapeutic proteins, such as IFN-β, enabling localized modulation of the TME [379]. Moreover, MSCs can be engineered to deliver oncolytic or immunomodulatory payloads to the TME, thereby enhancing CAR-T cell-mediated antitumor responses [380]. Collectively, these attributes position MSCs as a versatile biological vehicle for CAR-based strategies.

Recently, it was reported that genetic modification of MSCs with CD28-costimulated CAR enhances their antigen-specific immunosuppressive activity. The E-cadherin-targeted CAR-MSCs more effectively inhibited T cell responses and preferentially localized to E-cadherin-positive colonic epithelial cells, resulting in improved clinical symptoms and prolonged survival in animal models [17]. Although CAR-MSCs have demonstrated a favorable safety profile and show promise in the treatment of various immune-mediated and degenerative diseases, this field remains at an early developmental stage [377]. Significant challenges persist regarding their use as monotherapy or in combination with CAR-T cells and other immune-based therapies [22], underscoring the need for further mechanistic studies and translational investigation (Fig. 2C).

## Comparative considerations for different CAR cell platforms in distinct disease contexts

The expansion of CAR technology beyond conventional αβ T cells has given rise to a diverse set of cellular platforms with distinct biological features, manufacturing requirements, and clinical performance profiles. These platforms differ not only in cytotoxic potency and persistence, but also in safety profiles, manufacturability, scalability, and cost, precluding straightforward cross-platform comparisons (Fig. 1, Tables 1 and 2). Rigorous evaluation would ideally rely on head-to-head clinical studies conducted in matched disease settings; however, such data remain limited [159, 192, 239]. Nonetheless, emerging clinical and preclinical evidence supports context-dependent platform selection guided by disease biology and therapeutic objectives [161]. In hematologic malignancies, CAR-αβ T cells continue to represent the most effective modality for achieving deep and durable responses, whereas allogeneic alternatives, including CAR-γδ T, CAR-DNT, CAR-MAIT, CAR-NKT, and CAR-NK cells, offer favorable safety profiles and logistical advantages for patients requiring rapid treatment. In solid tumors, where inadequate trafficking and immunosuppressive TME limit T cell efficacy, CAR-M, CAR-T9, CAR-neutrophils, CAR-DCs, CAR-NKT, and CAR-γδ T cells exhibit enhanced tissue infiltration or microenvironmental modulation. Autoimmune diseases benefit from diverse CAR-based strategies that either eliminate or suppress pathogenic cells. While B cell-depleting CAR-αβ T cells have demonstrated remarkable efficacy in achieving clinical remission by eliminating pathogenic B cell clones, immunoregulatory platforms, such as CAR-Tregs and CAR-MSCs, enable antigen-specific immune suppression with reduced systemic toxicity [160]. Regarding manufacturing optimization and large-scale production, CAR-HSC and CAR-iPSC platforms provide transformative advantages. These sources enable the generation of standardized, clinical-grade “off-the-shelf” products, particularly for effector cell types that are rare or difficult to isolate from peripheral blood (Fig. 2A). Importantly, increasing attention is being directed toward multi-platform combination strategies that exploit complementary mechanisms, highlighting the need for rational integration rather than singular platform optimization (Fig. 2C, D) [381].

## Platform-specific optimization strategies and future directions

### Manufacturing innovations

To reduce manufacturing complexity and cost while improving production safety, continued innovation in CAR cell manufacturing is essential. Distinct CAR-engineered cell types exhibit fundamentally different biological properties and expansion requirements, necessitating cell type-specific optimization of manufacturing workflows. The development of universal, allogeneic CAR cell products represents a major future direction, as it enables standardized production, improved scalability, and broader clinical accessibility. In this context, stem cell-derived platforms, particularly iPSC-based CAR products, offer unique advantages, as banked iPSCs can serve as an essentially unlimited and renewable cell source capable of differentiation into multiple effector lineages. As manufacturing processes mature and “off-the-shelf” products replace autologous approaches, the cost of CAR-based cell therapies is expected to decline substantially, thereby facilitating wider clinical adoption (Fig. 2A). To date, all approved CAR-T cell products are manufactured *ex vivo*. This process requires isolation of patient-derived immune cells, genetic modification, and *ex vivo* expansion under controlled laboratory conditions. Consequently, it is associated with prolonged manufacturing timelines, the need for lymphodepleting conditioning, and substantial cost. Direct *in vivo* engineering of immune cells has emerged as a compelling alternative strategy that may overcome these limitations (Fig. 2A). Direct *in vivo* gene transfer using viral or non-viral platforms enables CAR expression in endogenous immune cells, thereby bypassing complex *ex vivo* manufacturing steps. Beyond T cells, *in vivo* CAR engineering approaches have also been explored in other immune cell types [65]. Notably, these strategies are being investigated across a broad range of indications, encompassing both malignant and non-malignant diseases [382]. Nevertheless, *in vivo* CAR engineering remains in its early stages. The efficiency of *in situ* cellular reprogramming, along with subsequent expansion and long-term persistence, requires substantial optimization. Moreover, the risks of off-target transduction and infusion-related toxicities must be rigorously assessed to ensure safety and clinical feasibility [65].

### CAR design innovations

Because activation and signaling requirements differ substantially across immune cell types, next-generation CAR designs should incorporate costimulatory modules optimized for specific cellular contexts, thereby tailoring effector functions toward either enhanced cytotoxicity or immunosuppression depending on disease indications. Target selection is central to the success of CAR therapies, as it fundamentally influences both efficacy and safety. Conventional CAR-T cells require tumor-restricted targets because their potent, long-lived cytotoxicity can cause severe on-target, off-tumor toxicity. In contrast, CAR-Tregs are designed to promote immune suppression and tolerance by targeting autoantigens or alloantigens and therefore do not require strict specificity. One example is CAR-Tregs targeting an alloantigen, such as HLA-A2, which is broadly expressed in various tissues and cells [175]. CAR-NK cells, which exhibit shorter persistence and rapid killing, may safely target shared tumor antigens that would be too risky for conventional CAR-T cells. Similarly, CAR-M preferentially engage antigens within the TME, leveraging phagocytosis and antigen cross–presentation [309]. Collectively, these examples illustrate that optimal antigen selection must be tailored to each cell type’s functional profile and therapeutic objective, highlighting the importance of matching CAR design to both cellular biology and disease context (Fig. 2B). Nevertheless, like CAR-T cells, CAR engineering across diverse platforms faces several shared limitations, including insufficient persistence, restricted tissue infiltration, antigen heterogeneity, and safety concerns. Addressing these challenges requires cell type-specific optimization strategies that align CAR architecture with intrinsic cellular biology. For example, the incorporation of synthetic receptor systems (such as synNotch), multi-antigen targeting strategies, light- or ultra-sensitive CARs [383, 384], and logic-gated CAR designs can improve specificity and safety by restricting activation to defined antigen combinations. In parallel, engineering approaches involving chemokine receptors or cytokine expression can enhance CAR cell persistence, functional fitness, and infiltration into target tissues [385]. Together, these design innovations provide a versatile framework for refining CAR performance across distinct cellular platforms and disease contexts (Fig. 2B).

### Combination strategies

The diverse CAR cell platforms and therapeutic modalities possess distinct properties, and their combination holds significant potential to enhance immunotherapy efficacy. Beyond integrating CAR cell therapy with oncolytic viruses [107], bacteria [386], vaccines, chemotherapy or radiotherapy, small-molecule inhibitors, and immune checkpoint inhibitors [105], combinatorial strategies increasingly include multiple CAR platforms or CAR cells paired with other cell-based therapies (Fig. 2C). In this review, we highlight combinations such as CAR-T with CAR-DCs or CAR-MSCs, as well as the complex interactions arising from CAR-HSC-derived multilineage CAR cells *in vivo*. Such combinatorial approaches have the potential to elicit stronger and more effective antitumor or disease-modifying responses.

### Disease expansion

CAR-T cell therapy has already demonstrated encouraging efficacy in hematologic malignancies and certain autoimmune diseases, driving the expansion of CAR approaches into non-cancer indications. CAR engineering now extends beyond conventional T cells, enabling treatment of a broader range of diseases and expanding therapeutic applications. Different diseases present variable burden and treatment windows, and multiple CAR cell platforms offer the flexibility to identify the most suitable cellular modality for each disease context (Fig. 2D).

## Conclusions

This review provides an overview of current CAR cell therapy platforms, encompassing their properties, advantages, sources, manufacturing processes, progress, challenges, and optimization strategies. Additionally, a comparative analysis of 13 CAR cell therapy platforms at both preclinical and clinical stages highlights their respective strengths and limitations. Although CAR–T cells remain the gold standard, their inherent limitations restrict applications in certain solid tumors and other disease contexts. This has catalyzed the expansion into 12 other diverse CAR-engineered cell types described in this review, which offer the potential for improved safety profiles without compromising efficacy, as well as the feasibility of “off-the-shelf” CAR-engineered therapeutics tailored to specific diseases.

In this review, we discuss 13 carrier cell types as living therapeutics; however, the CAR paradigm is not limited to these cells or even to living cells. Emerging platforms, such as CAR-engineered exosomes, represent a promising therapeutic modality [387], and emerging universal *in vivo* CAR technologies are poised to further revolutionize the future of cell therapy [67]. Additionally, CAR-engineered B cells and innate lymphoid cells are under early-stage development [388, 389], highlighting the potential for future expansion of CAR technologies to an even broader spectrum of cellular chassis. Collectively, these advances suggest that the CAR concept could extend far beyond its current scope, encompassing diverse cell types and modalities to address a wide range of diseases.

However, this expanding landscape of CAR technologies also introduces increasing complexity in how these platforms are evaluated and translated clinically. Different CAR cell platforms are typically evaluated under distinct disease contexts, making direct comparisons of therapeutic efficacy across cell types inherently challenging [390]. Although CAR-T cell therapy provides the most mature clinical framework and informs the development of other modalities, alternative CAR-engineered cell types possess lineage-specific biological properties that may shape their persistence, toxicity profiles, and functional behavior *in vivo*. With the exception of CAR-T cell therapy, although most platforms have undergone clinical testing, they remain in early stages of development and have not yet received approval from the U.S. FDA, resulting in substantial uncertainty and limited long-term follow-up data, despite the central importance of longitudinal monitoring for assessing memory effects, unintended toxicities, and immune persistence [42]. Accordingly, cross-platform CAR cell development requires lineage-specific preclinical and clinical evaluation, coupled with systematic long-term surveillance. Ethical assessment must likewise remain adaptive to platform-specific risks [391]. For example, CAR-engineered iPSCs raise concerns regarding long-term genomic stability and off-target gene editing [225], whereas *in vivo* CAR cell generation, despite its potential to improve accessibility and streamline manufacturing, introduces distinct translational risks, including unintended cell targeting, off-target transduction, and insertional mutagenesis [65].

At the same time, these challenges are shaping the next phase of innovation in CAR technologies. Increasing efforts are focused on developing more precisely controlled and programmable systems to enhance safety, functionality, and equitable access to therapy [392]. Moreover, the integration of multi-gene editing and cutting-edge technologies can construct sophisticated cellular programming systems to overcome current challenges in cell therapy, yielding novel optimization strategies for next-generation CAR therapeutics [393, 394]. These optimized CAR therapies can also be combined or used synergistically with other approaches to more effectively harness collective immune responses, thereby enhancing therapeutic efficacy against solid tumors and other diseases.
